# Utilization of clinical practice guidelines and interprofessional collaboration in depression management in Swiss primary care: a cross-sectional survey study among primary care physicians

**DOI:** 10.1186/s12875-025-02897-9

**Published:** 2025-07-02

**Authors:** Karin Mayer, Christoph Merlo, Stefan Markun, Stefan Neuner-Jehle, Patrick E. Beeler

**Affiliations:** 1 Center for Primary and Community Care, Faculty of Health Sciences and Medicine, University of Lucerne, Lucerne, Switzerland; 2 Institute of Primary Care, University Hospital Zurich, University of Zurich, Zurich, Switzerland; 3 Federal Office of Public Health (FOPH), Swiss Sentinel Surveillance System, Berne, Switzerland

**Keywords:** Depressive Disorder/diagnosis, Depressive Disorder/therapy, Anxiety, Primary Health Care, Practice Guidelines as Topic, Health Knowledge, Attitudes, Practice, Practice Patterns, Physicians', Guideline Adherence/standards, Cooperative Behavior, Switzerland, Cross-Sectional Studies

## Abstract

**Objectives:**

To (i) investigate the current state of depression management in Swiss primary care post-COVID-19, focusing on the utilization of guidelines or associated tools, (ii) explore potential associations with depression management, and (iii) evaluate availability of and communication with psychiatrists and psychotherapists.

**Methods:**

A previously developed self-report questionnaire, covering screening, diagnosis, management, and interprofessional collaboration, was distributed online, with a supplementary paper version, to 168 Swiss primary care physicians (PCPs) participating in the Swiss Sentinel Surveillance System. Multivariable logistic regressions explored associations.

**Results:**

Of the 168 primary care physicians invited to participate, 116 completed the survey (response rate: 69%). Among these, 61% utilized guidelines for depression management, with statistically significant associations towards increased guideline utilization for PCPs with some psychiatric training as residents (odds ratio [OR] 4.13; 95% confidence interval (95% CI) 1.27, 16.02) and decreased utilization for the age group 60–81 (OR 0.29; 95% CI 0.09, 0.93). Guideline utilization was associated with perceived competency in prescribing antidepressants (OR 3.51; 95% CI 1.21, 11.08). The majority reported difficulties in organizing therapy with mental health specialists and rarely received feedback after patient referrals.

**Conclusion:**

While the utilization of guidelines among participants was inconsistent, over 60% indicated using either guidelines, tools, or both. The study highlights systemic issues in interprofessional collaboration between PCPs and mental health specialists that require attention.

**Supplementary Information:**

The online version contains supplementary material available at 10.1186/s12875-025-02897-9.

## Introduction

From 1990 to 2019, mental disorders consistently ranked as the second leading cause of years lived with disability (YLDs) globally [1]. Across sex and year (1990- 2019), depressive and anxiety disorders emerge as the foremost mental health challenges, with depressive disorders claiming the highest rank within all age groups from 14 years and older [1].

The COVID-19 pandemic further exacerbated the global prevalence of depressive and anxiety disorders [2]. The 2022 Swiss Health Survey reveals a significant increase in mental health problems since the pandemic's onset, with the number of affected young people more than doubling, particularly elevating the number of moderately and severely psychologically distressed young women aged 15–24 [3, 4].

Primary care settings, responsible for 60% of mental health care delivery [5], play a crucial role in managing depression, estimated to have a prevalence of 30% in Swiss primary care physicians’ (PCPs) consultations [6]. However, deficits in the quality of depression management within the primary care sector have been reported by numerous studies [5] and are reflected in the global untreated rate of approximately 56.3% among individuals with depression [7]. In Switzerland, 27% of individuals experiencing mental health problems do not seek any form of help [8]. This not only underscores the importance of patient education, health literacy, and self-management but also the potential value of screenings – a topic of ongoing debate with diverging recommendations [9].

Clinical Practice Guidelines (CPGs) provide recommendations for the screening, diagnosis, and treatment of depression, but their content and quality of reporting vary significantly [10]. American and European guidelines make similar recommendations regarding the treatment of depression based on severity and comorbidities [11]. In Switzerland, three-quarters of consultations for depression reveal comorbidities [12], complicating treatment and worsening prognoses.

Despite the importance of guidelines in improving mental health care, guideline adherence remains inconsistent across Europe. According to a recent study conducted in the Zurich region, only 15% of general practitioners (GPs) are using guidelines when facing a patient with a potential depressive disorder [13], while in Germany, 65% of PCPs reported never or rarely using the national S3 Guideline for Unipolar Depression [14, 15]. Similarly, in France, less than a third of PCPs were aware of depression guidelines, with many relying on their own judgment rather than adhering to guideline criteria [16, 17].

Several potential barriers have been identified to hinder physician adherence to guidelines, including lack of awareness, familiarity, confidence in ability to implement the guidelines (self-efficacy), agreement with the recommendations, and belief in positive outcomes from following the guidelines (outcome expectancy) [18].

Treatment strategies for depression as outlined in guidelines typically involve psychotherapy along pharmacotherapy, necessitating collaborative efforts between PCPs and mental health professionals, such as psychologists or psychiatrists [11, 19]. Reports indicate that such interprofessional collaboration is often suboptimal in the Swiss healthcare system [20].

In response to the documented challenges in depression management within Swiss primary care, the present study was conducted as the first of its kind within the Swiss context following the COVID-19 pandemic. Given the significant disruptions in healthcare during the pandemic, including changes in care delivery and increased mental health needs [2–4, 21], it is important to consider whether and how these factors may be associated with the use of clinical guidelines in depression management. While previous research has explored the impact of the pandemic on mental healthcare utilization [21], no studies specifically examined whether these disruptions relate to the current use of clinical guidelines and tools for managing depression in primary care. This knowledge gap is relevant as the pandemic has likely altered the capacity for and the approach to depression care.

Understanding that effective provision is the foundation of comprehensive depression management, and that provision is guided by evidence-based guidelines, the study primarily aimed to assess the current state of provision by investigating the utilization of guidelines and associated tools for depression screening and diagnosis in nation-wide Swiss primary care practices. The study additionally aimed to explore physicians’ depression knowledge, specifically examining their relationship to the use of clinical guidelines and tools, as well as the availability of and communication with psychiatrists and psychotherapists within the healthcare system.

To achieve these objectives, a cross-sectional survey was distributed to 168 Swiss PCPs who are actively engaged in the Swiss Sentinel Surveillance System.

## Methods

### Study design, setting and participants

This cross-sectional survey study was conducted within the Swiss Sentinel Surveillance System (Sentinella) between March 30 and November 14, 2023. The Sentinella Network comprises voluntarily engaged PCPs, including general practitioners, internists, and pediatricians, collaborating with the Federal Office of Public Health (FOPH). This collaboration monitors communicable acute diseases and conducts research in general practice medicine on a national level [22]. The survey was distributed to all 168 active reporting units of the Sentinella Network, excluding those on extended reporting breaks. No additional exclusion criteria were applied.

The study reporting adhered to the Checklist for Reporting of Survey Studies (CROSS) [23], which can be found in the appendix (Table 21).

### Ethical considerations

The FOPH manages the administrative aspects of the Swiss Sentinel Surveillance System and ensures the inclusion of general practitioners distributed across all regions of Switzerland to maintain overall representativeness. All communication and administration of the present study were conducted through the FOPH, providing anonymity of the participants and confidentiality.

Participants were informed about the study's objectives through a cover letter accompanying the survey. An electronic consent paragraph was included before initiating the survey, explaining that participation was voluntary and that by participating, individuals agreed to their responses being analyzed and published in an anonymized form.

### Data collection methods

Data was collected through convenience single-stage sampling, employing a questionnaire specifically developed for this study. The questionnaire design was informed by group discussions with Swiss PCPs and the Sentinella program commission and took into consideration relevant literature on depression management within the primary care setting. Available in German and French, the questionnaire was initially distributed using Google Forms and later supplemented with a paper version. Participants received the online survey link via e-mail on March 30, 2023, with reminders sent one and three months later. To enhance representativeness, the sample size aimed for was 60% of the 168 reporting units, equivalent to 100 participants. To reach this rate, a third reminder in the form of a paper survey, was sent mid-November 2023.

Comprising 33 questions across five parts, the questionnaire covered 1) information on medical practice, 2) approach, 3) management, 4) therapy, and 5) interprofessional collaboration, all in the context of depression in the primary care setting. Questions employed closed-ended multiple-choice formats, including self-defined answer options, pre-existing dichotomous answers, and five-point Likert scale responses, where score 1 was the lowest or negative response anchor and score 5 the highest or positive response anchor. There were three additional free-text questions. The first question was mandatory and required participants to provide their Sentinella identification number, which helped prevent multiple participation and link the responses to demographic data. The second question asked participants to specify the guidelines used, if any, and the third provided participants with the option to give feedback at the end of the survey. Apart from these latter two questions, all responses were mandatory in the online survey, while the paper version did not have the capability to enforce mandatory answers. The original questionnaires are provided in appendix Table 22 and Table 23, with an English version available in appendix Table 24.

Demographic data was not directly collected but provided for each Sentinella identification number through the FOPH and included sex, age group and information on practice location (urban/intermediate/rural, as well as region by Sentinella classification).

### Statistical analysis

Statistical analyses were performed using R, version 4.2.2. For descriptive analyses, categorical variables are presented as counts and percentages, with Chi-square tests comparing groups. Continuous variables with non-normal distributions are depicted as medians and interquartile ranges (IQR) and comparisons were made using Mann–Whitney-U and Kruskal–Wallis tests. Data visualization plots were generated using the *likert* package (version 1.3.5).

Open-ended feedback items were individually analyzed and coded, and major themes were summarized according to their frequencies [24].

The utilization of guidelines and corresponding tools (for screening and diagnosis) was the primary focus of logistic multivariable regression models, reporting odds ratios (ORs) and 95% confidence intervals (95% CIs). This binary variable was a composite derived from responses to three survey questions asking: 1) if guidelines were used for depression (and/or suicidality) diagnosis and/or management, 2) if systematic depression screening was conducted for each patient, and 3) how depression was diagnosed. While the first two questions had yes/no answers, the third question offered a range of response options, including a predefined selection of tools, “Others”,"Without specific diagnostic means"and"If there is any suspicion, I refer these patients". Participants were categorized into a group that uses guidelines and/or corresponding tools if they selected"Yes"for question 1 and/or 2, and/or indicated the use of at least one tool or"Others"for question 3. Conversely, the group that does not use guidelines or corresponding tools comprised participants who selected"No"for question 1 and 2, and indicated"If there is any suspicion, I refer these patients"and/or"Without specific diagnostic means"for question 3.

In a first regression model, the use of guidelines and corresponding tools served as outcome, whereas various variables were tested for potential associations with this outcome. Further multivariable regression models investigated the use of guidelines and corresponding tools as exposure for different outcomes related to PCPs’ knowledge and management of depression in primary care.

Multiple imputation using the R package *mice* (version 3.16.0) [25] addressed missing demographic elements. This method was applied to four demographic variables: age group, sex, practice location (urban/rural/intermediate), and region by Sentinella classification.

Missing data primarily stemmed from challenges in linking the demographic information to corresponding identification numbers. This occurred particularly in cases where a reporting unit comprised multiple (up to three) PCPs, with varying demographic details, practicing within the same practice. Additionally, discrepancies in identification numbers and gaps in demographic data provided by the FOPH further contributed to the presence of missing or unmatchable data.

## Results

### Characteristics of participants

A total of 116 PCPs participated in the survey, with 86 respondents completing it online and 30 utilizing the paper version. This yielded a response rate of 69%. Table 1 outlines the demographic characteristics of participants, stratified by their utilization or non-utilization of guidelines and corresponding tools. Additional stratifications are provided in the appendix (Table 7- 12).

**Table 1:** Participant characteristics by utilization of guidelines and corresponding tools

	**Non-utilization of guidelines and tools**	**Utilization of guidelines and tools**	**p**	**Overall**
**N**	45	71		116
**Age group (%)**			0.104	
Aged 35–49	9 (20.0)	27 (38.0)		36 (31.0)
Aged 50–59	13 (28.9)	22 (31.0)		35 (30.2)
Aged 60–81	16 (35.6)	17 (23.9)		33 (28.4)
Unknown	7 (15.6)	5 (7.0)		12 (10.3)
**Sex (%)**			0.583	
Female	13 (28.9)	25 (35.2)		38 (32.8)
Male	25 (55.6)	39 (54.9)		64 (55.2)
Unknown	7 (15.6)	7 (9.9)		14 (12.1)
**Paper survey (%)**			0.352	
No	36 (80.0)	50 (70.4)		86 (74.1)
Yes	9 (20.0)	21 (29.6)		30 (25.9)
**Urban or rural area (%)**			0.283	
Urban	27 (62.8)	54 (76.1)		81 (69.8)
Intermediate	10 (23.3)	11 (16.2)		21 (18.1)
Rural	6 (14.0)	4 (5.9)		10 (8.6)
Unknown	2 (4.4)	2 (2.8)		4 (3.4)
^a^**Region by Sentinella classification (%)**			0.059	
Southwestern Switzerland	8 (18.6)	24 (33.8)		32 (27.6)
Northeastern Switzerland	11 (25.6)	11 (16.2)		22 (19.0)
Western Swiss Plateau	4 (9.3)	17 (25.0)		21 (18.1)
Northwestern Switzerland	8 (18.6)	8 (11.8)		16 (13.8)
Southeastern Switzerland	7 (16.3)	7 (10.3)		14 (12.1)
Central Switzerland	5 (11.6)	2 (2.9)		7 (6.0)
Unknown	2 (4.4)	2 (2.8)		4 (3.4)
**Dispensation of medication (%)**			0.112	
No	19 (42.2)	42 (59.2)		61 (52.6)
Yes	26 (57.8)	29 (40.8)		55 (47.4)
**Participation in Balint groups (%)**			0.454	
No	41 (91.1)	60 (84.5)		101 (87.1)
Yes	4 (8.9)	11 (15.5)		15 (12.9)
**Psychiatric training as resident (%)**			0.138	
No	39 (86.7)	52 (73.2)		91 (78.4)
Yes	6 (13.3)	19 (26.8)		25 (21.6)
^b^**SAPPM certificate (%)**			0.284	
No	43 (95.6)	62 (87.3)		105 (90.5)
No, but I'm on the way to obtaining it	0 (0.0)	2 (2.8)		2 (1.7)
Yes	2 (4.4)	7 (9.9)		9 (7.8)

^a^Regional specifications can be found in Table 6 in the appendix

^b^Interdisciplinary focus on psychosomatic and psychosocial medicine

Missing data are attributed to non-matching Sentinella identification numbers (4 cases), and in one instance the age group was unknown. Additionally, data showed discrepancies for multiple PCPs registering under the same identification number, differing in age group (7 cases) or sex (10 cases).

### Utilization of guidelines and corresponding tools

Among the 116 participants, 25 reported using guidelines for the assessment and/or management of depressed patients and/or suicidality (appendix Table 1). Furthermore, of 116 participants, five reported conducting systematic, standardized depression screenings for each patient in their practice. When asked about their approach to diagnosing depression, most participants indicated that they did not use any specific diagnostic tools, as evidenced in Table 2.

**Table 2:** How participants diagnose depression

Guidelines or tools	N responses (%)
Without special diagnostic tools (%)	61 (52.6)
In case of suspicion, I refer these patients (%)	31 (26.7)
Diagnostic Manual (ICD-10, ICD-11, DSM-5) (%)	31 (26.7)
Hamilton Scale (HS) (%)	22 (19.0)
Beck Depression Inventory (BDI) (%)	12 (10.3)
PHQ-9 (%)	9 (7.8)
Aphasic Depression Rating Scale (ADRS)^a^ (%)	3 (2.6)
PHQ-2 (%)	2 (1.7)
Montgomery–Åsberg Depression Rating Scale (MADRS)^a^ (%)	1 (0.9)
Others (%)	7 (6.0)

^a^from free-text responses

Primary regression analysis encompassed all 116 responses. It investigated associations between guideline and tool utilization and characteristics of participants (appendix Table 13). Findings revealed a statistically significant association towards decreased guideline/tool utilization for the age group 60–81, with an OR of 0.29 (95% CI 0.09, 0.93). Conversely, those who completed some psychiatric training as residents were statistically significantly associated to more likely indicate the utilization of guidelines and tools (OR 4.13; 95% CI 1.27, 16.02). Compared to the region Northwestern Switzerland used as the reference, the regions Western Swiss Plateau (OR 9.35; 95% CI 1.73, 62.61) and Southwestern Switzerland (OR 5.27; 95% CI 1.20, 25.29) were associated with higher utilization rates.

### Knowledge and management

**Fig. 1:**
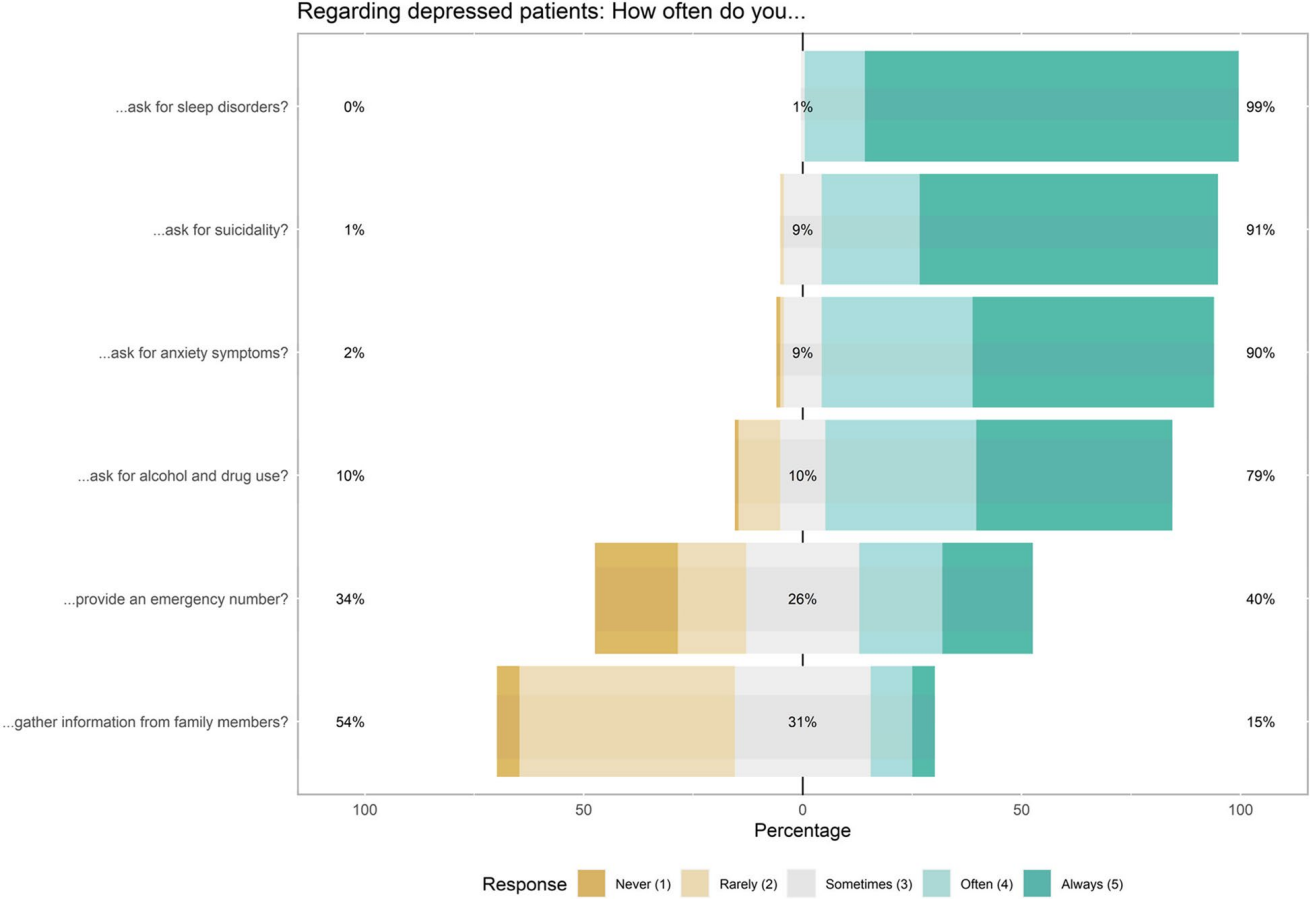
Participants answer distributions regarding depression knowledge and management

Figure 1, found below, illustrates that a substantial majority of PCPs (ranging from 79–99%) reported always or often inquiring about sleep disorders, suicidality, anxiety symptoms, and substance use in depressed patients. Of the participants, 40% stated that they always or often provided emergency contact numbers to their patients, and 15% reported always or often gathering information from family members of depressed patients.

Table 3 demonstrates that three-quarters of PCPs consider distinguishing bipolar disorder from depression as important. and that the majority expressed confidence in providing primary care talk therapy and prescribing antidepressant medication therapy.

**Table 3:** Participants answer distributions regarding depression knowledge and management

Question	Mean	Median	Statistical mode (peak)	N responses	Percent of total	Response	N responses	Proportion of N (%)
How important is it to you to differentiate depression from bipolar disorder?	4.12	Rather important (4)	Very important (5)	115	99.1	Completely unimportant (1)	0	0
						Rather unimportant (2)	1	0.9
						Neutral (3)	28	24.4
						Rather important (4)	42	36.5
						Very important (5)	44	38.3
In primary care talk therapy (00.0520 Psychotherapeutic/ psychosocial counseling by primary care specialist, per 5 min.) I feel…	3.59	Rather competent (4)	Rather competent (4)	116	100	Very incompetent (1)	2	1.7
						Rather incompetent (2)	7	6
						Neutral (3)	39	33.6
						Rather competent (4)	56	48.3
						Very competent (5)	12	10.3
In prescribing antidepressant medication therapy I feel…	3.44	Rather competent (4)	Rather competent (4)	116	100	Very incompetent (1)	4	3.5
						Rather incompetent (2)	12	10.3
						Neutral (3)	37	31.9
						Rather competent (4)	55	47.4
						Very competent (5)	8	6.9

Appendix Tables 2, 3 and 4 provide insight into participants responses concerning the rationales for prescribing psychotropic drugs, referring patients to psychotherapy, and the urgent hospitalization of depressed patients, respectively.

Exploratory regression analysis was conducted to uncover associations with variables related to the knowledge and management of depression, specifically examining their relationship with the utilization of guidelines and corresponding tools (exposure). Results indicated a significant association, with PCPs who utilized guidelines and tools being more likely to feel very or rather competent in prescribing antidepressant medication therapy (OR 3.51; 95% CI 1.21, 11.08; cf. appendix Table 16).

Regression results further revealed that PCPs who had completed some psychiatric training as residents were significantly associated to more likely feel very or rather competent in primary care talk therapy (OR 6.4; 95% CI 2.02, 25.25; cf. appendix Table 17).

Additional regression analysis investigating the collection of information from family members of depressed patients, revealed a statistically significant association indicating that PCPs who self-dispensed medication were less inclined to report that they always or often collected information from family members (OR 0.23; 95% CI 0.06, 0.76; cf. appendix Table 18).

### Therapy & interprofessional collaboration

**Fig. 2:**
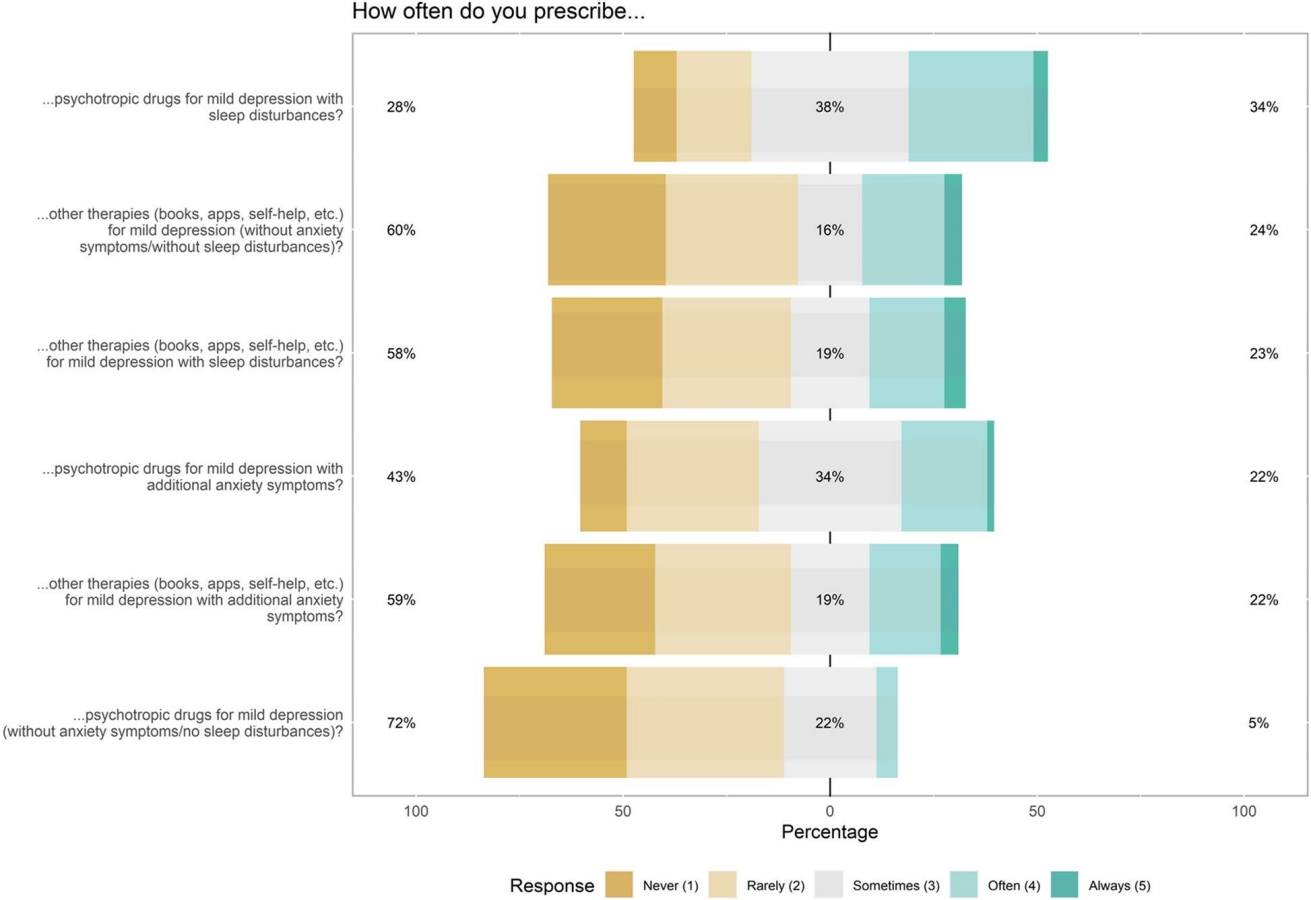
Participants answer distributions regarding depression therapy

Figure 2 provides information on the treatment preferences of PCPs for mild depression. Notably, participants displayed a tendency to prescribe psychotropic drugs more frequently for mildly depressed patients presenting symptoms of anxiety or sleep disturbances, compared to those without additional symptoms.

Concerning the availability of and communication with psychotherapists and psychiatrists, Table 4 reveals perceived challenges in organizing therapy places. Regarding interprofessional communication, PCPs, in general, rarely received feedback from psychotherapists and psychiatrists after referring a patient. Further insights into how PCPs received feedback versus their preferred feedback methods are detailed in Table 2 in the appendix.

**Table 4:** Participants answer distributions regarding availability of and communication with psychotherapists and psychiatrists

Question	Mean	Median	Statistical mode (peak)	N responses	Percent of total (%)	Responses	N responses	Proportion of N (%)
Availability of therapy places with psychological psychotherapists: How easy or difficult is it to organize a therapy place?	2.22	Rather difficult, large time investment (2)	Rather difficult, large time investment (2)	116	100	Very difficult, very large time investment (1)	30	25.9
						Rather difficult, large time investment (2)	51	44
						Neutral (3)	16	13.8
						Rather easy, sometimes with a little time investment (4)	18	15.5
						Very easy (5)	1	0.9
Availability of therapy places with psychiatrists: How easy or difficult is it to organize a therapy place?	1.72	Very difficult, very large time investment (1)	Very difficult, very large time investment (1)	116	100	Very difficult, very large time investment (1)	61	52.6
						Rather difficult, large time investment (2)	35	30.2
						Neutral (3)	11	9.5
						Rather easy, sometimes with a little time investment (4)	9	7.8
						Very easy (5)	0	0
How often do you receive feedback from psychological psychotherapists after referrals of depressed patients?	2.41	Rarely (21–40%) (2)	Rarely (21–40%) (2)	116	100	Never (0–20%) (1)	19	16.4
						Rarely (21–40%) (2)	48	41.4
						Sometimes (41–60%) (3)	33	28.5
						Often (61–80%) (4)	14	12.1
						Always (81–100%) (5)	2	1.7
How often do you receive feedback from psychiatrists after referrals of depressed patients?	2.38	Rarely (21–40%) (2)	Rarely (21–40%) (2)	116	100	Never (0–20%) (1)	27	23.3
						Rarely (21–40%) (2)	38	32.8
						Sometimes (41–60%) (3)	33	28.5
						Often (61–80%) (4)	16	13.8
						Always (81–100%) (5)	2	1.7

Regression analysis revealed that participants who felt competent in prescribing antidepressant medication therapy (OR 3.88; 95% CI 1.61, 9.97) and participants of the age group 60–81 (OR 4.44; 95% CI 1.46, 14.86) were statistically significantly associated to more often prescribing psychotropic drugs for mild depression, with or without additional symptoms (appendix Table 14).

No associations were found between prescription of self-help therapy for mild depressions and the utilization of guidelines and tools.

Regarding alternative (self-help) therapy prescription, male participants (OR 0.39; 95% CI 0.15, 0.99) and participants in the age group 50–59 (OR 0.32; 95% CI 0.09, 0.99) were statistically significantly associated to less likely report always or often prescribing alternative therapy for mild depression with or without additional symptoms (appendix Table 15).

### Feedback section

Appendix Table 19 and Table 20 offer insights into participants’ feedback responses and major identified themes. Participants predominantly expressed concerns highlighting the need for changes in the healthcare system (*N* = 6). Two participants specifically mentioned the lack of time resources for comprehensive and adequate therapy of depressed patients. Five participants addressed interprofessional collaboration with psychotherapists and psychiatrists, while two participants noted an improvement in communication with psychotherapists since the implementation of new regulations for psychological psychotherapy of July 1, 2022. These regulations allow psychotherapists to work independently and bill their services through the compulsory health insurance, given a medical prescription [26].

## Discussion

### Summary of main findings

The study revealed that approximately 60% of PCPs engaged in either utilizing guidelines or tools for diagnosing or screening depression. Although significant regional differences were observed, no significant differences were found between urban and rural settings. On the one hand, the age group 60–81 was statistically significantly associated with the absence of utilization. On the other hand, psychiatric training as a resident was associated with the utilization. Additionally, there was a significant association between the utilization of guidelines and tools and increased perceived competency in prescription of antidepressant medication therapy. No other associations were identified to suggest that PCPs using guidelines or tools have superior management approaches.

### Comparison with existing literature

The study’s findings that only 25 PCPs reported using guidelines for the management of depressed patients (appendix Table 1) corresponds with the findings of the study in the Swiss German region, revealing that only 15% of GPs used guidelines for mild depression management [13]. These findings are also consistent with guideline utilization in other European countries, such as Germany and France, where guideline adherence is similarly low [14–17].

Another Swiss study revealed that while physicians generally had favorable attitudes towards guidelines and believed they could improve quality of care, only one third reported frequent guideline use, due to barriers such as lack of awareness/familiarity and time constraints [27]. Therefore, the fact that PCPs of the present study with some prior psychiatric training showed higher rates of guideline and tool utilization suggests a greater awareness and familiarity with depression guidelines, due to exposure during their training.

Contrary associations with the age group 60–81 of PCPs exhibiting lower rates of guideline utilization are relatable to results of another study revealing that physicians who have been in practice longer may be less likely to adhere to standards of practice regarding screening and diagnosis [28].

The present study found a low rate of systematic and standardized depression screenings among PCPs. There is an ongoing debate regarding the prevalence of underdiagnosis or overdiagnosis of depression, with conflicting evidence in the literature [29–34], resulting in diverging recommendations regarding depression screenings [9]. The US Preventive Services Task Force guidelines recommend routine depression screening for adult patients in primary care settings, citing evidence of moderate benefit in improving health outcomes [35]. However, European guidelines, including Swiss guidelines (MediX, Mednet) cited by participants recommend a more targeted approach to depression screening, focusing on at-risk patients [14, 36–38]. In the US, routine depression screenings are billed using a specific procedure code (96127) defined by the American Medical Association's Current Procedural Terminology (CPT) [39], which facilitates their implementation in practice. Switzerland lacks a standardized billing system for such screenings, which may pose challenges for their implementation.

Another association between the age group 60–81 of PCPs and higher rates in frequency of psychotropic drug prescription for mild depression supports the findings of a Swiss study suggesting that longer practical experience of general practitioners (GPs) is associated with medication overprescription [13].

Many studies from European countries, including Switzerland [40], show overprescription of antidepressants in treatment practices [16, 34, 41, 42]. A French study among GPs found that medication was cited as the most frequently used treatment followed by psychotherapy and cognitive behavioral therapy (CBT), and almost never self-help literature [16], despite recommendations [36]. Additionally, a German study found that approximately 60% of the depressed patients were not being treated as recommended in the national guidelines [43].

On the one hand, the present study’s findings on mild depression support the observation that alternative therapies, such as self-help interventions, although recommended by guidelines, are rarely prescribed – perhaps due to a perceived lack of clinical efficacy stemming from insufficient evidence supporting their effectiveness [44]. On the other hand, findings concerning the prescription of psychotropic drugs align with results of a Swiss study conducted by Hengartner et al. [13], which found that PCPs would rarely recommend antidepressants in mild depression with no comorbidities, while the presence of sleep problems or anxiety symptoms increased the likelihood of antidepressant prescription.

Contrary to the findings of Hengartner et al. [13], the present study did not find an independent, statistically significant association between self-dispensation of medication and the prescription of antidepressants. Instead, it suggests that perceived competency in prescribing antidepressant medication therapy may have a greater influence on the actual prescription of antidepressants.

Moreover, the results revealing an association between the utilization of guidelines and tools and increased competency in prescribing antidepressant medication imply that guidelines may comprehensively cover antidepressant prescription practices. However, it is essential to consider that individual PCP characteristics influence this subjective competency, and actual medication prescription practices may still deviate from guideline recommendations.

Notably, irrespective of utilizing guidelines and tools, participants'responses in many cases aligned with European guidelines. The majority recognized the importance of distinguishing between bipolar and depressive disorder and inquired about suicidality, anxiety, sleep disorders, and substance abuse in depressed patients, as recommended [14, 36–38].

This study observed considerable difficulties in organizing therapy places with psychiatrists and psychotherapists and the infrequent feedback following patient referrals. These difficulties resonate with research from Germany, revealing that only 22.1% of PCPs reported good cooperation with mental health specialists [15], and from France, where PCPs similarly reported obstacles in accessing mental healthcare services and obtaining timely and satisfactory responses from specialists [16].

Lack of time has emerged as a major obstacle to providing adequate care for depressed patients, be it among PCPs or mental health specialists. PCPs have identified it as a key reason for referring patients to psychotherapy and have also highlighted the substantial time investment required for organizing therapy appointments with psychotherapists and psychiatrists, suggesting that health specialists may also experience time constraints.

### Limitations

The interpretation of results and its implications must consider the following limitations of the study.

As the study's questionnaire was not based on a validated instrument, the reliability and accuracy of the captured practices and attitudes could potentially be affected. Still, the questionnaire was informed by PCPs themselves in collaboration with the Sentinella program commission. This collaboration involved specialists experienced in research methodologies.

Furthermore, the study was limited in sample size and may therefore not be sufficiently representative. This may have contributed to the study's inability to detect statistically significant associations compared to others [13]. The study, however, achieved a high response rate within the sample group, exceeding the targeted 60%.

The fact that participants were drawn from a pool of voluntarily engaged physicians may have introduced selection bias, possibly skewing the sample towards a more engaged subset. Consequently, the findings may not be generalizable to the broader PCP population. Nevertheless, utilizing the Swiss Sentinella Network facilitated access to nationwide, systematic data collection.

Furthermore, responses may be subject to self-reporting bias, with participants providing socially desirable responses deviating from their actual practice. Yet, Sentinella network participants, given their voluntarily participation specifically for research purposes, are known to report in a professional manner.

Although querying demographic information through the FOPH using identification numbers ensured anonymity, it resulted in a notable amount of missing data in demographic variables. This issue could have been prevented by directly inquiring the information in the survey. Missing data, however, were addressed by means of multiple imputations for regression analyses.

Moreover, the study does not delve into underlying motivations behind utilization of guidelines and tools and associations in depression management. For instance, actual practices in therapy management could be influenced by patient-centered approach and shared decision-making. Although participants implied these factors when asked about reasons for management decisions (e.g. “patient request”), the study did not investigate them further. However, the underlying motivations of PCPs were beyond the scope of the study.

In addition, the study did not include an objective counterpart, such as reviewing clinical records or conducting patient interviews, to objectively validate the findings, which would have helped to ensure that reported practices reflect actual clinical behavior. As this was beyond the scope of the study, future research could integrate these objective measures to validate and complement the findings [43].

Lastly, also being outside of the scope of the study, the role of physical exercise as a management tool for depression was not addressed, despite evidence showing its significant effectiveness in reducing depressive symptoms [45]. Including questions related to physical exercise would have added depth to the study.

Despite these limitations, the study's mixed-mode survey approach, with both paper and online versions, increased reach and probability of capturing physicians with diverse characteristics, therefore enhancing the credibility of the findings.

Overall, this study as the first of its kind within Switzerland post-COVID-19, provides valuable insights into the utilization of guidelines and corresponding tools, depression management, and collaboration with psychiatrists and psychotherapists among Swiss PCPs that can inform future research and clinical practice.

### Implications

The association between the age group 60–81 of PCPs and decreased guideline and tool utilization, as well as increased psychotropic drugs prescription rates for mild depression, may imply that younger PCPs have successfully been reached by evidence-based depression management approaches, possibly because medical education in Switzerland has already adapted to evidence-based medicine. The link to education is further reinforced by the study’s findings that PCPs who completed some psychiatric training as residents were associated with higher guideline and tool utilization rates and increased feelings of competency in primary care talk therapy. This is supported by literature revealing that, likely due to their more extensive training in this area, younger GPs exhibited more positive attitudes towards guideline utilization [46]. Mercier et al. [16] furthermore revealed that trained GPs were much more comfortable coping with depressed patients, less frequently using secondary care providers, and preferred alternative solutions rather than antidepressant drugs. These findings highlight the importance of medical education in preparing PCPs to effectively manage depression and suggest that integrating practical training in evidence-based guidelines into medical education programs may be beneficial.

In light of the identified regional variations in guidelines and tool utilization, further investigation into educational practices across the different regions could reveal potential factors contributing to the observed differences. Specifically, examining whether certain regions incorporate guidelines and tool utilization into their educational programs may provide insights into the disparities in utilization rates observed.

Depression management in Switzerland might furthermore benefit of the development of updated and unified evidence-based guidelines tailored to the Swiss context. This could help standardize practices and overcome barriers to implementation, similar to the approach taken in Germany with their S3 guidelines. Moreover, involving PCPs in the development of guidelines from an early stage could ensure their successful implementation and usage in clinical practice [15]. This is because such guidelines may closely align with the everyday challenges and experiences faced by PCPs, and early involvement could cultivate a greater sense of confidence and ownership/identification with the guidelines.

Finally, the findings suggest systemic issues within the healthcare system regarding collaboration between PCPs, psychotherapists, and psychiatrists that require attention. While limited improvements have been noted since July 2022, more changes appear necessary to enhance collaboration and ensure comprehensive depression management in primary care settings.

## Conclusion

To achieve comprehensive depression management in Swiss primary care settings, systemic changes within the health care system are necessary. These changes may involve regulatory adjustments, enhancements in the renumeration system, strengthening the workforce of both PCPs and mental health specialists, and supporting new models of interprofessional collaboration and managed care. Primary care in Switzerland could furthermore benefit from the development of unified and updated evidence-based depression guidelines, tailored to the Swiss context, and medical education could be an important contributor to guideline implementation.

## Supplementary Information

Supplemenary Material 1.

## Data Availability

The datasets used and analyzed during the current study are available from the corresponding author on reasonable request.
