# Supplementary material for: Utilization of clinical practice guidelines and interprofessional collaboration in depression management in Swiss primary care: a cross-sectional survey study among primary care physicians
Source: BMC Prim Care. 2025 Jul 2;26:210. doi: 10.1186/s12875-025-02897-9 (PMC12217389; doi:10.1186/s12875-025-02897-9)
Supplement: Supplementary file 1 — Supplemenary Material 1. [file 12875_2025_2897_MOESM1_ESM.docx]

Appendix

Supplementary Table 1: Guidelines specified for the assessment and/or management of depressed patients and/or suicidality

| **Guidelines** | **N responses (%)** |
| --- | --- |
| MediX Guidelines (%) | 6 (24.0) |
| Mednet Guidelines (%) | 3 (12.0) |
| Montgomery–Åsberg Depression Rating Scale (MADRS) (%) | 1 (8.0) |
| MADRS or Beck Depression Inventory (BDI) (%) | 1 (4.0) |
| ICD-10 (%) or DSM-4 (%) | 1 (4.0) |
| Others (%) | 13 (52.0) |

Other guidelines included unspecified guidelines from predecessors or education, strategies of cantonal hospitals, evidence based medicine guidelines (EbM) and uptodate.com.

Supplementary Table 2: PCPs answers regarding reason(s) for referral of depressed patients to psychotherapy

| **Reasons** | **N responses (%)** |
| --- | --- |
| Severity of depression (%) | 100 (86.2) |
| Patient request (%) | 88 (75.9) |
| Lack of time (%) | 54 (46.6) |
| For own emotional relief (%) | 25 (21.6) |
| Lack of specialized competence (%)^a^ | 8 (6.9) |
| Lack of interest (%) | 7 (6.0) |
| Labour law and insurance considerations (%)^a^ | 6 (5.2) |
| Other reasons (unspecified) (%) | 10 (8.6) |

# ^a^ from free-text responses

Supplementary Table 3: PCPs answers regarding reason(s) for psychotropic drugs prescription for depressed patients

| **Reasons** | **N responses (%)** |
| --- | --- |
| Severity of depression (%) | 95 (81.9) |
| Accompanying symptoms such as anxiety or sleep disorders (%) | 78 (67.2) |
| Patient request (%) | 55 (47.4) |
| Lack of time (%) | 2 (1.7) |
| I do not prescribe psychotropic drugs (%)^a^ | 2 (1.7) |
| Other reasons (unspecified) (%) | 6 (5.2) |

# ^a^ from free-text responses

Supplementary Table 4: PCPs answers regarding most common reason(s) for an urgent psychiatric hospitalization of depressed patients

**N responses (%)**

**Reasons**

| Self-endangerment/acute suicidality (%) | 107 (92.2) |
| --- | --- |
| Severity of depression (%) | 63 (54.3) |
| Patient request (%) | 60 (51.7) |
| Lack of response to previous treatment (%) | 39 (33.6) |
| Psychiatric comorbidity, e.g. addiction problems (%) | 31 (26.7) |
| Request of relatives (%) | 26 (22.4) |
| Social circumstances (%)^a^ | 2 (1.7) |
| Other reasons (unspecified) (%) | 1 (0.9) |

# ^a^ from free-text responses

Supplementary Table 5: In what form participants mostly receive vs. would like to receive feedback from psychotherapists/psychiatrists on the diagnosis and/or the course of therapy

|  | **Effective form of feedback** | **Desired form of feedback** |
| --- | --- | --- |
| **Form** | **N responses (%)** | **N responses (%)** |
| E-Mail (%) | 59 (50.9) | 76 (65.5) |
| Phone (%) | 34 (29.3) | 17 (14.7) |
| Post letter (%) | 29 (25.0) | 36 (31.0) |
| Other (%) | 3 (2.6) | 1 (0.9) |

Supplementary Table 6: Regional specifications

| **Region by Sentinella Classification** | **Cantons** |
| --- | --- |
| Northwestern Switzerland | Geneva (GE), Neuchâtel (NE), Vaud (VD),  Valais (VS) |
| Northeastern Switzerland | Appenzell Innerrhoden (AI), Appenzell Ausserrhoden (AR), Glarus (GL), St.Gallen (SG), Schaffhausen (SH), Thurgau (TG),  Zurich (ZH) |
| Western Swiss Plateau | Berne (BE), Fribourg (FR), Jura (JU) |
| Southwestern Switzerland | Aargau (AG), Basel-Country (BL), Basel-  City (BS), Solothurn (SO) |
| Southeastern Switzerland | Graubünden (GR), Ticino (TI) |
| Central Switzerland | Lucerne (LU), Nidwalden (NW), Obwalden  (OW), Schwyz (SZ), Uri (UR), Zug (ZG) |

Supplementary Table 7: Participant characteristics by guideline use

|  | **No** | **Yes** | **p** | **Overall** |
| --- | --- | --- | --- | --- |
| **N** | 91 | 25 |  | 116 |
| **Age group (%)** |  |  | 0.356 |  |
| Aged 35-49 | 25 (27.5) | 11 (44.0) |  | 36 (31.0) |
| Aged 50-59 | 28 (30.8) | 7 (28.0) |  | 35 (30.2) |
| Aged 60-81 | 27 (29.7) | 6 (24.0) |  | 33 (28.4) |
| Unknown | 11 (12.1) | 1 (4.0) |  | 12 (10.3) |
| **Sex (%)** |  |  | 0.327 |  |
| Female | 28 (30.8) | 10 (40.0) |  | 38 (32.8) |
| Male | 50 (54.9) | 14 (56.0) |  | 64 (55.2) |
| Unknown | 13 (14.3) | 1 (4.0) |  | 14 (12.1) |
| **Paper survey (%)** |  |  | 0.126 |  |
| No | 64 (70.3) | 22 (88.0) |  | 86 (74.1) |
| Yes | 27 (29.7) | 3 (12.0) |  | 30 (25.9) |
| **Region (Sentinella Classification) (%)** |  |  | 0.042* |  |
| Northwestern Switzerland | 14 (15.4) | 2 (8.0) |  | 16 (13.8) |
| Northeastern Switzerland | 18 (19.8) | 4 (16.0) |  | 22 (19.0) |
| Western Swiss Plateau | 11 (12.1) | 10 (40.0) |  | 21 (18.1) |
| Southwestern Switzerland | 25 (27.5) | 7 (28.0) |  | 32 (27.6) |
| Southeastern Switzerland | 13 (14.3) | 1 (4.0) |  | 14 (12.1) |
| Central Switzerland | 7 (7.7) | 0 (0.0) |  | 7 (6.0) |
| Unknown | 3 (3.3) | 1 (4.0) |  | 4 (3.4) |
| **Urban or rural area (%)** |  |  | 0.51 |  |
| Intermediate | 19 (20.9) | 2 (8.0) |  | 21 (18.1) |
| Rural | 8 (8.8) | 2 (8.0) |  | 10 (8.6) |
| Urban | 61 (67.0) | 20 (80.0) |  | 81 (69.8) |
| Unknown | 3 (3.3) | 1 (4.0) |  | 4 (3.4) |
| **Dispensation of medication (%)** |  |  | 0.541 |  |
| No | 46 (50.5) | 15 (60.0) |  | 61 (52.6) |
| Yes | 45 (49.5) | 10 (40.0) |  | 55 (47.4) |
| **Participation in Balint groups (%)** |  |  | 0.857 |  |
| No | 80 (87.9) | 21 (84.0) |  | 101 (87.1) |
| Yes | 11 (12.1) | 4 (16.0) |  | 15 (12.9) |
| **Psychiatric training as resident (%)** |  |  | 0.541 |  |
| No | 73 (80.2) | 18 (72.0) |  | 91 (78.4) |
| Yes | 18 (19.8) | 7 (28.0) |  | 25 (21.6) |
| **SAPPM certificate (%)** |  |  | 0.001* |  |
| No | 87 (95.6) | 18 (72.0) |  | 105 (90.5) |
| No, but I'm on the way to obtaining it | 0 (0.0) | 2 (8.0) |  | 2 (1.7) |
| Yes | 4 (4.4) | 5 (20.0) |  | 9 (7.8) |

Supplementary Table 8: Participant characteristics by systematic depression screening

|  | **No** | **Yes** | **p** | **Overall** |
| --- | --- | --- | --- | --- |
| **N** | 111 | 5 |  | 116 |
| **Age group (%)** |  |  | 0.431 |  |
| Aged 35-48 | 35 (31.5) | 1 (20.0) |  | 36 (31.0) |
| Aged 50-58 | 34 (30.6) | 1 (20.0) |  | 35 (30.2) |
| Aged 60-80 | 30 (27.0) | 3 (60.0) |  | 33 (28.4) |
| Unknown | 12 (10.8) | 0 (0.0) |  | 12 (10.3) |
| **Sex (%)** |  |  | 0.479 |  |
| Female | 37 (33.3) | 1 (20.0) |  | 38 (32.8) |
| Male | 60 (54.1) | 4 (80.0) |  | 64 (55.2) |
| Unknown | 14 (12.6) | 0 (0.0) |  | 14 (12.1) |
| **Paper survey (%)** |  |  | 0.829 |  |
| No | 83 (74.8) | 3 (60.0) |  | 86 (74.1) |
| Yes | 28 (25.2) | 2 (40.0) |  | 30 (25.9) |
| **Region (Sentinella classification) (%)** |  |  | 0.684 |  |
| Northwestern Switzerland | 16 (14.4) | 0 (0.0) |  | 16 (13.8) |
| Northeastern Switzerland | 20 (18.0) | 2 (40.0) |  | 22 (19.0) |
| Western Swiss Plateau | 21 (18.9) | 0 (0.0) |  | 21 (18.1) |
| Southwestern Switzerland | 30 (27.0) | 2 (40.0) |  | 32 (27.6) |
| Southeastern Switzerland | 13 (11.7) | 1 (20.0) |  | 14 (12.1) |
| Central Switzerland | 7 (6.3) | 0 (0.0) |  | 7 (6.0) |
| Unknown | 4 (3.6) | 0 (0.0) |  | 4 (3.4) |
| **Urban or rural area (%)** |  |  | 0.389 |  |
| Intermediate | 19 (17.1) | 2 (40.0) |  | 21 (18.1) |
| Rural | 9 (8.1) | 1 (20.0) |  | 10 (8.6) |
| Urban | 79 (71.2) | 2 (40.0) |  | 81 (69.8) |
| Unknown | 4 (3.6) | 0 (0.0) |  | 4 (3.4) |
| **Dispensation of medication (%)** |  |  | 1 |  |
| No | 58 (52.3) | 3 (60.0) |  | 61 (52.6) |
| Yes | 53 (47.7) | 2 (40.0) |  | 55 (47.4) |
| **Participation in Balint groups (%)** |  |  | 1 |  |
| No | 97 (87.4) | 4 (80.0) |  | 101 (87.1) |
| Yes | 14 (12.6) | 1 (20.0) |  | 15 (12.9) |
| **Psychiatric training as resident (%)** |  |  | 0.639 |  |
| No | 88 (79.3) | 3 (60.0) |  | 91 (78.4) |
| Yes | 23 (20.7) | 2 (40.0) |  | 25 (21.6) |
| **SAPPM certificate (%)** |  |  | 0.005* |  |
| No | 101 (91.0) | 4 (80.0) |  | 105 (90.5) |
| No, but I'm on the way to obtaining it | 1 (0.9) | 1 (20.0) |  | 2 (1.7) |
| Yes | 9 (8.1) | 0 (0.0) |  | 9 (7.8) |

Supplementary Table 9 Participant characteristics by psychiatric training as resident

|  | **No** | **Yes** | **p** | **Overall** |
| --- | --- | --- | --- | --- |
| **N** | 91 | 25 |  | 116 |
| **Age group (%)** |  |  | 0.287 |  |
| Aged 35-48 | 30 (33.0) | 6 (24.0) |  | 36 (31.0) |
| Aged 50-58 | 24 (26.4) | 11 (44.0) |  | 35 (30.2) |
| Aged 60-80 | 26 (28.6) | 7 (28.0) |  | 33 (28.4) |
| Unknown | 11 (12.1) | 1 (4.0) |  | 12 (10.3) |
| **Sex (%)** |  |  | 0.923 |  |
| Female | 29 (31.9) | 9 (36.0) |  | 38 (32.8) |
| Male | 51 (56.0) | 13 (52.0) |  | 64 (55.2) |
| Unknown | 11 (12.1) | 3 (12.0) |  | 14 (12.1) |
| **Paper survey (%)** |  |  | 0.619 |  |
| No | 66 (72.5) | 20 (80.0) |  | 86 (74.1) |
| Yes | 25 (27.5) | 5 (20.0) |  | 30 (25.9) |
| **Region (Sentinella classification) (%)** |  |  | 0.978 |  |
| Northwestern Switzerland | 13 (14.3) | 3 (12.0) |  | 16 (13.8) |
| Northeastern Switzerland | 18 (19.8) | 4 (16.0) |  | 22 (19.0) |
| Western Swiss Plateau | 16 (17.6) | 5 (20.0) |  | 21 (18.1) |
| Southwestern Switzerland | 24 (26.4) | 8 (32.0) |  | 32 (27.6) |
| Southeastern Switzerland | 12 (13.2) | 2 (8.0) |  | 14 (12.1) |
| Central Switzerland | 5 (5.5) | 2 (8.0) |  | 7 (6.0) |
| Unknown | 3 (3.3) | 1 (4.0) |  | 4 (3.4) |
| **Urban or rural area (%)** |  |  |  |  |
| Intermediate | 13 (14.3) | 8 (32.0) | 0.199 | 21 (18.1) |
| Rural | 9 (9.9) | 1 (4.0) |  | 10 (8.6) |
| Urban | 66 (72.5) | 15 (60.0) |  | 81 (69.8) |
| Unknown | 3 (3.3) | 1 (4.0) |  | 4 (3.4) |
| **Dispensation of medication (%)** |  |  | 0.873 |  |
| No | 47 (51.6) | 14 (56.0) |  | 61 (52.6) |
| Yes | 44 (48.4) | 11 (44.0) |  | 55 (47.4) |
| **Participation in Balint groups (%)** |  |  | 0.622 |  |
| No | 78 (85.7) | 23 (92.0) |  | 101 (87.1) |
| Yes | 13 (14.3) | 2 (8.0) |  | 15 (12.9) |
| **SAPPM certificate (%)** |  |  | 0.539 |  |
| No | 81 (89.0) | 24 (96.0) |  | 105 (90.5) |
| No, but I'm on the way to obtaining it | 2 (2.2) | 0 (0.0) |  | 2 (1.7) |
| Yes | 8 (8.8) | 1 (4.0) |  | 9 (7.8) |

Supplementary Table 10: Participant characteristics by participation in Balint groups

|  | **No** | **Yes** | **p** | **Overall** |
| --- | --- | --- | --- | --- |
| **N** | 101 | 15 |  | 116 |
| **Age group (%)** |  |  | 0.36 |  |
| Aged 35-49 | 33 (32.7) | 3 (20.0) |  | 36 (31.0) |
| Aged 50-59 | 29 (28.7) | 6 (40.0) |  | 35 (30.2) |
| Aged 60-81 | 30 (29.7) | 3 (20.0) |  | 33 (28.4) |
| Unknown | 9 (8.9) | 3 (20.0) |  | 12 (10.3) |
| **Sex (%)** |  |  | 0.388 |  |
| Female | 32 (31.7) | 6 (40.0) |  | 38 (32.8) |
| Male | 58 (57.4) | 6 (40.0) |  | 64 (55.2) |
| Unknown | 11 (10.9) | 3 (20.0) |  | 14 (12.1) |
| **Paper survey (%)** |  |  | 0.695 |  |
| No | 76 (75.2) | 10 (66.7) |  | 86 (74.1) |
| Yes | 25 (24.8) | 5 (33.3) |  | 30 (25.9) |
| **Region (Sentinella Classification) (%)** | 14 (13.9) | 2 (13.3) | 0.304 |  |
| Northwestern Switzerland | 20 (19.8) | 2 (13.3) |  | 16 (13.8) |
| Northeastern Switzerland | 18 (17.8) | 3 (20.0) |  | 22 (19.0) |
| Western Swiss Plateau | 18 (17.8) | 3 (20.0) |  | 21 (18.1) |
| Southwestern Switzerland | 29 (28.7) | 3 (20.0) |  | 32 (27.6) |
| Southeastern Switzerland | 13 (12.9) | 1 (6.7) |  | 14 (12.1) |
| Central Switzerland | 4 (4.0) | 3 (20.0) |  | 7 (6.0) |
| Unknown | 3 (3.0) | 1 (6.7) |  | 4 (3.4) |
| **Urban or rural area (%)** |  |  | 0.48 |  |
| Intermediate | 19 (18.8) | 2 (13.3) |  | 21 (18.1) |
| Rural | 10 (9.9) | 0 (0.0) |  | 10 (8.6) |
| Urban | 69 (68.3) | 12 (80.0) |  | 81 (69.8) |
| Unknown | 3 (3.0) | 1 (6.7) |  | 4 (3.4) |
| **Dispensation of medication (%)** |  |  | 0.83 |  |
| No | 54 (53.5) | 7 (46.7) |  | 61 (52.6) |
| Yes | 47 (46.5) | 8 (53.3) |  | 55 (47.4) |
| **Psychiatric training as resident (%)** |  |  | 0.622 |  |
| No | 78 (77.2) | 13 (86.7) |  | 91 (78.4) |
| Yes | 23 (22.8) | 2 (13.3) |  | 25 (21.6) |
| **SAPPM certificate (%)** |  |  | <0.001* |  |
| No | 96 (95.0) | 9 (60.0) |  | 105 (90.5) |
| No, but I'm on the way to obtaining it | 0 (0.0) | 2 (13.3) |  | 2 (1.7) |
| Yes | 5 (5.0) | 4 (26.7) |  | 9 (7.8) |

Supplementary Table 11: Participant characteristics by medication dispensation

|  | **No** | **Yes** | **p** | **Overall** |
| --- | --- | --- | --- | --- |
| **N** | 61 | 55 |  | 116 |
| **Age group (%)** |  |  | 0.025* |  |
| Aged 35-49 | 24 (39.3) | 12 (21.8) |  | 36 (31.0) |
| Aged 50-59 | 19 (31.1) | 16 (29.1) |  | 35 (30.2) |
| Aged 60-81 | 16 (26.2) | 17 (30.9) |  | 33 (28.4) |
| Unknown | 2 (3.3) | 10 (18.2) |  | 12 (10.3) |
| **Sex (%)** |  |  | 0.017* |  |
| Female | 25 (41.0) | 13 (23.6) |  | 38 (32.8) |
| Male | 33 (54.1) | 31 (56.4) |  | 64 (55.2) |
| Unknown | 3 (4.9) | 11 (20.0) |  | 14 (12.1) |
| **Paper survey (%)** |  |  | 0.758 |  |
| No | 44 (72.1) | 42 (76.4) |  | 86 (74.1) |
| Yes | 17 (27.9) | 13 (23.6) |  | 30 (25.9) |
| **Region (Sentinella classification) (%)** |  |  | <0.001* |  |
| Northwestern Switzerland | 11 (18.0) | 5 (9.1) |  | 16 (13.8) |
| Northeastern Switzerland | 1 (1.6) | 21 (38.2) |  | 22 (19.0) |
| Western Swiss Plateau | 11 (18.0) | 10 (18.2) |  | 21 (18.1) |
| Southwestern Switzerland | 27 (44.3) | 5 (9.1) |  | 32 (27.6) |
| Southeastern Switzerland | 11 (18.0) | 3 (5.5) |  | 14 (12.1) |
| Central Switzerland | 0 (0.0) | 7 (12.7) |  | 7 (6.0) |
| Unknown | 0 (0.0) | 4 (7.3) |  | 4 (3.4) |
| **Urban or rural area (%)** |  |  | 0.1 |  |
| Intermediate | 10 (16.4) | 11 (20.0) |  | 21 (18.1) |
| Rural | 4 (6.6) | 6 (10.9) |  | 10 (8.6) |
| Urban | 46 (75.4) | 34 (61.8) |  | 81 (69.8) |
| Unknown | 1 (1.6) | 4 (7.3) |  | 4 (3.4) |
| **Participation in Balint groups (%)** |  |  | 0.83 |  |
| No | 54 (88.5) | 47 (85.5) |  | 101 (87.1) |
| Yes | 7 (11.5) | 8 (14.5) |  | 15 (12.9) |
| **Psychiatric training as resident (%)** |  |  | 0.873 |  |
| No | 47 (77.0) | 44 (80.0) |  | 91 (78.4) |
| Yes | 14 (23.0) | 11 (20.0) |  | 25 (21.6) |
| **SAPPM certificate (%)** |  |  | 0.981 |  |
| No | 55 (90.2) | 50 (90.9) |  | 105 (90.5) |
| No, but I'm on the way to obtaining it | 1 (1.6) | 1 (1.8) |  | 2 (1.7) |
| Yes | 5 (8.2) | 4 (7.3) |  | 9 (7.8) |

Supplementary Table 12: Participant characteristics by SAPPM certificate

|  | **No** | **No, but I'm on the way to**  **obtaining it** | **Yes** | **p** | **Overall** |
| --- | --- | --- | --- | --- | --- |
| **N** | 105 | 2 | 9 |  | 116 |
| **Age group (%)** |  |  |  | 0.562 |  |
| Aged 35-48 | 35 (33.3) | 0 (0.0) | 1 (11.1) |  | 36 (31.0) |
| Aged 50-58 | 30 (28.6) | 1 (50.0) | 4 (44.4) |  | 35 (30.2) |
| Aged 60-80 | 30 (28.6) | 1 (50.0) | 2 (22.2) |  | 33 (28.4) |
| Unknown | 10 (9.5) | 0 (0.0) | 2 (22.2) |  | 12 (10.3) |
| **Sex (%)** |  |  |  | 0.896 |  |
| Female | 33 (31.4) | 1 (50.0) | 4 (44.4) |  | 38 (32.8) |
| Male | 59 (56.2) | 1 (50.0) | 4 (44.4) |  | 64 (55.2) |
| Unknown | 13 (12.4) | 0 (0.0) | 1 (11.1) |  | 14 (12.1) |
| **Paper survey (%)** |  |  |  | 0.619 |  |
| No | 78 (74.3) | 2 (100.0) | 6 (66.7) |  | 86 (74.1) |
| Yes | 27 (25.7) | 0 (0.0) | 3 (33.3) |  | 30 (25.9) |
| **Region (Sentinella classification) (%)** |  |  |  | 0.176 |  |
| Northwestern Switzerland | 16 (15.2) | 0 (0.0) | 0 (0.0) |  | 16 (13.8) |
| Northeastern Switzerland | 19 (18.1) | 2 (100.0) | 1 (11.1) |  | 22 (19.0) |
| Western Swiss Plateau | 17 (16.2) | 0 (0.0) | 4 (44.4) |  | 21 (18.1) |
| Southwestern Switzerland | 30 (28.6) | 0 (0.0) | 2 (22.2) |  | 32 (27.6) |
| Southeastern Switzerland | 13 (12.4) | 0 (0.0) | 1 (11.1) |  | 14 (12.1) |
| Central Switzerland | 7 (6.7) | 0 (0.0) | 0 (0.0) |  | 7 (6.0) |
| Unknown | 3 (2.9) | 0 (0.0) | 1 (11.1) |  | 4 (3.4) |
| **Urban or rural area (%)** |  |  |  | 0.819 |  |
| Intermediate | 19 (18.1) | 0 (0.0) | 2 (22.2) |  | 21 (18.1) |
| Rural | 9 (8.6) | 0 (0.0) | 1 (11.1) |  | 10 (8.6) |
| Urban | 74 (70.5) | 2 (100.0) | 5 (55.6) |  | 81 (69.8) |
| Unknown | 3 (2.9) | 0 (0.0) | 1 (11.1) |  | 4 (3.4) |
| **Dispensation of medication (%)** |  |  |  | 0.981 |  |
| No | 55 (52.4) | 1 (50.0) | 5 (55.6) |  | 61 (52.6) |
| Yes | 50 (47.6) | 1 (50.0) | 4 (44.4) |  | 55 (47.4) |
| **Participation in Balint groups (%)** |  |  |  | <0.001* |  |
| No | 96 (91.4) | 0 (0.0) | 5 (55.6) |  | 101 (87.1) |
| Yes | 9 (8.6) | 2 (100.0) | 4 (44.4) |  | 15 (12.9) |
| **Psychiatric training as resident (%)** |  |  |  | 0.539 |  |
| No | 81 (77.1) | 2 (100.0) | 8 (88.9) |  | 91 (78.4) |
| Yes | 24 (22.9) | 0 (0.0) | 1 (11.1) |  | 25 (21.6) |

Supplementary Table 13

# Outcome: Use of guidelines & corresponding tools

| **Variable** | **OR** | **(95% CI)** | **Stat. sign.** |
| --- | --- | --- | --- |
| Aged 35-49 | Ref |  |  |
| Aged 50-59 | 0.58 | (0.17,1.84) |  |
| Aged 60-81 | 0.29 | (0.09,0.93) | * |
| Female | Ref |  |  |
| Male | 2 | (0.74,5.71) |  |
| Urban Practice Location | Ref |  |  |
| Intermediate practice location | 0.18 | (0.05,0.62) | * |
| Rural practice location | 0.53 | (0.10,2.74) |  |
| Northwestern Switzerland | Ref |  |  |
| Northeastern Switzerland | 3.18 | (0.65,17.24) |  |
| Western Swiss Plateau | 9.35 | (1.73,62.61) | * |
| Southwestern Switzerland | 5.27 | (1.20,25.29) | * |
| Southeastern Switzerland | 1.6 | (0.33,7.98) |  |
| Central Switzerland | 0.32 | (0.02,3.33) |  |
| Dispensation of medication | Ref |  |  |
| No dispensation of medication | 0.65 | (0.21,1.98) |  |
| No participation in Balint groups | Ref |  |  |
| Participation in Balint groups | 2.58 | (0.56,14.88) |  |
| No psychiatric training as resident | Ref |  |  |
| Psychiatric training as resident | 4.13 | (1.27,16.02) | * |
| No SAPPM certificate | Ref |  |  |
| SAPPM certificate^a^ | 1.94 | (0.36,14.98) |  |

^a^ Including those on the way to obtaining the certificate

OR: odds ratio

95%CI: 95% confidence interval Stat. sign.: statistically significant Ref: reference=1

Supplementary Table 14

Outcome: Often or always prescribe medication therapy for mild depression with or without additional symptoms

| **Variable** | **OR** | **(95% CI)** | **Stat. sign.** |
| --- | --- | --- | --- |
| No use of guidelines & tools | Ref |  |  |
| Use of guidelines & tools | 1.26 | (0.49,3.28) |  |
| No dispensation of medication | Ref |  |  |
| Dispensation of medication | 1.3 | (0.56,3.04) |  |
| Feel neutral or incompetent in medication therapy | Ref |  |  |
| Feel competent in medication therapy | 3.88 | (1.61,9.97) | * |
| Aged 35-49 | Ref |  |  |
| Aged 50-59 | 2.7 | (0.88,8.95) |  |
| Aged 60-81 | 4.44 | (1.46,14.86) | * |
| Female | Ref |  |  |
| Male | 1.21 | (0.49,3.04) |  |
| Urban Practice Location | Ref |  |  |
| Intermediate practice location | 1.97 | (0.60,6.63) |  |
| Rural practice location | 0.58 | (0.10,2.73) |  |
| No participation in Balint groups | Ref |  |  |
| Participation in Balint groups | 2.89 | (0.79,11.44) |  |
| No psychiatric training as resident | Ref |  |  |
| Psychiatric training as resident | 0.51 | (0.15,1.59) |  |
| No SAPPM certificate | Ref |  |  |
| SAPPM certificate^a^ | 0.87 | (0.18,4.09) |  |

^a^ Including those on the way to obtaining the certificate

OR: odds ratio

95%CI: 95% confidence interval Stat. sign.: statistically significant Ref: reference=1

Supplementary Table 15

# Outcome: Often or always prescribe alternative (self-help) therapy for mild depression with or without additional symptoms

| **Variable** | **OR** | **(95% CI)** | **Stat. sign.** |
| --- | --- | --- | --- |
| No use of guidelines & tools | Ref |  |  |
| Use of guidelines & tools | 0.66 | (0.25,1.72) |  |
| No dispensation of medication | Ref |  |  |
| Dispensation of medication | 1.04 | (0.41,2.61) |  |
| Feel neutral or incompetent in medication therapy | Ref |  |  |
| Feel competent in medication therapy | 1.43 | (0.58,3.64) |  |
| Aged 35-49 | Ref |  |  |
| Aged 50-59 | 0.32 | (0.09,0.99) | * |
| Aged 60-81 | 0.48 | (0.16,1.40) |  |
| Female | Ref |  |  |
| Male | 0.39 | (0.15,0.99) | * |
| Urban Practice Location | Ref |  |  |
| Intermediate practice location | 0.52 | (0.14,1.69) |  |
| Rural practice location | 1.54 | (0.29,6.80) |  |
| No participation in Balint groups | Ref |  |  |
| Particpation in Balint groups | 0.71 | (0.14,2.96) |  |
| No psychiatric training as resident | Ref |  |  |
| Psychiatric training as resident | 1.18 | (0.35,3.69) |  |
| No SAPPM certificate | Ref |  |  |
| SAPPM certificate^a^ | 0.73 | (0.09,3.92) |  |

^a^ Including those on the way to obtaining the certificate

OR: odds ratio

95%CI: 95% confidence interval Stat. sign.: statistically significant Ref: reference=1

Supplementary Table 16

# Outcome: Perceived competence in medication therapy (very & rather competent)

| **Variable** | **OR** | **(95% CI)** | **Stat. sign.** |
| --- | --- | --- | --- |
| No use of guidelines & tools | Ref |  |  |
| Use of guidelines & tools | 3.51 | (1.21,11.08) | * |
| No dispensation of medication | Ref |  |  |
| Dispensation of medication | 2.03 | (0.76,5.75) |  |
| Feel neutral or incompetent in talk therapy | Ref |  |  |
| Feel competent in talk therapy | 23.91 | (8.05,85.14) | * |
| Aged 35-49 | Ref |  |  |
| Aged 50-59 | 1.26 | (0.36,4.64) |  |
| Aged 60-81 | 1.46 | (0.43,5.07) |  |
| Female | Ref |  |  |
| Male | 2.34 | (0.85,6.93) |  |
| Urban Practice Location | Ref |  |  |
| Intermediate practice location | 0.77 | (0.21,2.82) |  |
| Rural practice location | 0.69 | (0.12,4.08) |  |
| No participation in Balint groups | Ref |  |  |
| Particpation in Balint groups | 0.52 | (0.09,3.03) |  |
| No psychiatric training as resident | Ref |  |  |
| Psychiatric training as resident | 0.5 | (0.13,1.88) |  |
| No SAPPM certificate | Ref |  |  |
| SAPPM certificate^a^ | 3.78 | (0.50,48.75) |  |

^a^ Including those on the way to obtaining the certificate

OR: odds ratio

95%CI: 95% confidence interval Stat. sign.: statistically significant Ref: reference=1

Supplementary Table 17

# Outcome: Perceived competence in talk therapy (very & rather competent)

| **Variable** | **OR** | **(95% CI)** | **Stat. sign.** |
| --- | --- | --- | --- |
| No use of guidelines & tools | Ref |  |  |
| Use of guidelines & tools | 0.82 | (0.33,1.97) |  |
| Aged 35-49 | Ref |  |  |
| Aged 50-59 | 0.56 | (0.19,1.60) |  |
| Aged 60-81 | 0.53 | (0.19,1.47) |  |
| Female | Ref |  |  |
| Male | 0.92 | (0.39,2.20) |  |
| Urban Practice Location | Ref |  |  |
| Intermediate practice location | 1.44 | (0.46,4.87) |  |
| Rural practice location | 1.06 | (0.24,4.60) |  |
| No dispensation of medication | Ref |  |  |
| Dispensation of medication | 0.98 | (0.43,2.23) |  |
| No participation in Balint groups | Ref |  |  |
| Particpation in Balint groups | 2.46 | (0.61,12.64) |  |
| No psychiatric training as resident | Ref |  |  |
| Psychiatric training as resident | 6.4 | (2.02,25.25) | * |
| No SAPPM certificate | Ref |  |  |
| SAPPM certificate^a^ | 3.67 | (0.69,28.91) |  |

^a^ Including those on the way to obtaining the certificate

OR: odds ratio

95%CI: 95% confidence interval Stat. sign.: statistically significant Ref: reference=1

Supplementary Table 18

# Outcome: Always or often collect information of family members

| **Variable** | **OR** | **(95% CI)** | **Stat. sign.** |
| --- | --- | --- | --- |
| No use of guidelines & tools | Ref |  |  |
| Use of guidelines & tools | 1.16 | (0.34,4.32) |  |
| Aged 35-49 | Ref |  |  |
| Aged 50-59 | 1.16 | (0.24,5.58) |  |
| Aged 60-81 | 2.9 | (0.71,13.54) |  |
| Female | Ref |  |  |
| Male | 2.22 | (0.66,8.97) |  |
| Urban Practice Location | Ref |  |  |
| Intermediate practice location | 1.34 | (0.25,6.10) |  |
| Rural practice location | 0 | (0.00,7.766742e+33) |  |
| No dispensation of medication | Ref |  |  |
| Dispensation of medication | 0.23 | (0.06,0.76) | * |
| No participation in Balint groups | Ref |  |  |
| Particpation in Balint groups | 0.22 | (0.01,2.09) |  |
| No psychiatric training as resident | Ref |  |  |
| Psychiatric training as resident | 0.59 | (0.11,2.42) |  |
| No SAPPM certificate | Ref |  |  |
| SAPPM certificate^a^ | 2.58 | (0.24,23.59) |  |

^a^ Including those on the way to obtaining the certificate

Supplementary Table 19: Feedback remarks of participants with major themes

| **Feedback remarks** | **Major themes** |
| --- | --- |
| Als Kinderärztin arbeite ich mit der Kinder- und Jugendpsychiatrie zusammen | Interprofessional Collaboration  Pediatricians |
| Äusserst dürftige Verfügbarkeit von psychologischem/psychiatrischem Fachpersonal, Wartezeit > 3 Monate. Zunehmender Druck seitens der Versicherungen und psychiatrischer Gutachter psychiatrisches/psychologisches Fachpersonal hinzu zu ziehen. Zunehmende Verweise der Gutachter, dass Grundversorger keine  adäquate Behandlung depressiver Personen übernehmen können. | Interprofessional Collaboration  Healthcare system |
| Avec le nouveau bon de psychologie délégué, le médecin de premier recours est plus au courant des problèmes psy de ses patients et cela amène et le patient et le psychologue à plus communiquer avec nous et améliore la compréhension des problèmes de nos patients. Néanmoins le feed-back du psy devrait être obligatoire avant la prescription de la  2ième série de séances | Regulation of July 2022  Interprofessional Collaboration  Healthcare system |
| Bei allen psychiatrischen Problemen ist die soziale Integration wichtig:  v.a. Mitglied in irgendwelchen Vereinen! Religiöse Bindungen sind sehr wertvoll, sofern nicht verkümmert. | Social Integration |

| Die gilt v.a. für die Integration von Migrantinnen, speziell infolge Heirat.  Deshalb hier Gründung der Stiftung «Mintegra» (Migration und Integration!) vor über 20 Jahren!" |  |
| --- | --- |
| Bessere Kommunikation durch Pyschotherapeuten seit 7/2022! | Interprofessional Collaboration Regulation of July  2022 |
| Bias: habe PsychologInnen in der Praxid und bin mit Psychiarin  verheiratet | Interprofessional  Collaboration |
| compte tenu de la grande fréquence et en augmentation des troubles  psy, je trouve que l'accès psy doit être libre, sans passer par le généraliste | Healthcare system |
| Die meisten Patientinnen werden aus stationärer Behandlung entlassen  ohne Anaschlussbehandlung!!! | Healthcare system |
| en pédiatrie, nous voyons des parents déprimés et l'impact sur les  enfants avec difficulté qu'ils se prennent en charge | Pediatricians |
| Frage 16) Ich verschreibe als Kinderarzt keine antidepressive  Pharmakatherapie | Pediatricians |
| La dépression est très chronophage... Je fais au mieux, mais manque de  disponibilité pour prise en charge complète ... | Time constraints |
| médecin praticien limité dans la perception des psychothérapies -  scandaleux! | Healthcare system |
| Pädiater - wenig «echte Depressionen» | Pediatricians |
| Pas de temps pour troubles psy tant que médecin praticien | Time constraints |
| Rechne mit 00.0525 ab nie 00.0520 | Healthcare system |

# Frequencies of major themes

| **N** | **15** |
| --- | --- |
| Healthcare System | 6 |
| Interprofessional Collaboration | 5 |
| Pediatricians | 4 |
| Time constraints | 2 |
| Regulation of July 2022 | 2 |
| Social Integration | 1 |

Supplementary Table 20: Feedback remarks of participants with major themes (English translation)

| **Feedback remarks** | **Major themes** |
| --- | --- |
| As a pediatrician, I collaborate with child and adolescent psychiatry. | Interprofessional  Collaboration Pediatricians |

| Extremely limited availability of psychological/psychiatric professionals, waiting time > 3 months. Increasing pressure from insurance companies and psychiatric experts to involve psychiatric/psychological professionals. Growing referrals from experts indicating that primary care providers cannot adequately treat  depressive individuals. | Interprofessional Collaboration  Healthcare system |
| --- | --- |
| With the new delegated psychology voucher, the primary care doctor is more aware of their patients' mental health issues, leading to more communication between the patient, psychologist, and us, and improving understanding of the patient's problems. Nevertheless, feedback from the psychologist should be mandatory before prescribing  the second series of sessions. | Regulation of July 2022  Interprofessional Collaboration  Healthcare system |
| For all psychiatric problems, social integration is important, especially being a member of any associations! Religious ties are very valuable, as long as they are not underdeveloped. This applies particularly to the integration of migrants, especially due to marriage. Therefore, the foundation "Mintegra" (Migration and Integration!) was established  more than 20 years ago! | Social Integration |
| Better communication through psychotherapists since July 2022! | Interprofessional Collaboration Regulation of July  2022 |
| Bias: I have psychologists in the practice and am married to a  psychiatrist. | Interprofessional  Collaboration |
| Given the high and increasing frequency of mental health disorders, I  believe that access to psychological services should be free, without needing to go through a general practitioner. | Healthcare system |
| Most patients are discharged from inpatient care without follow-up  treatment!!! | Healthcare system |
| In pediatrics, we see depressed parents and the impact on the children,  who struggle to take care of themselves. | Pediatricians |
| Question 16) I do not prescribe antidepressant medication as a  pediatrician. | Pediatricians |
| Depression is very time-consuming... I do my best, but there is not  enough time for complete care... | Time constraints |
| Practicing doctor is limited in their understanding of psychotherapies –  scandalous! | Healthcare system |
| Pediatrician – few "real" depressions. | Pediatricians |
| No time for psychiatric issues as a practicing doctor. | Time constraints |
| I charge with 00.0525, never 00.0520. | Healthcare system |

# Frequencies of major themes

| **N** | **15** |
| --- | --- |
| Healthcare System | 6 |
| Interprofessional Collaboration | 5 |

| Pediatricians | 4 |
| --- | --- |
| Time constraints | 2 |
| Regulation of July 2022 | 2 |
| Social Integration | 1 |

Supplementary Table 21: Checklist for Reporting Of Survey Studies (CROSS)

**Section/topic Item Item description Reported on page #**

**Title and abstract**

Title and abstract

1a State the word “survey” along with a commonly used term in title or abstract to introduce the study’s design.

Provide an informative summary in the abstract, covering background,

*1*

*1-2*

**Introduction**

1b objectives, methods, findings/results, interpretation/discussion, and conclusions.

Background 2 Provide a background about the rationale of study, what has been

previously done, and why this survey is needed.

*3-4*

Purpose/aim 3 Identify specific purposes, aims, goals, or objectives of the study. *4*

**Methods**

Study design 4 Specify the study design in the methods section with a commonly used *5*

term (e.g., cross-sectional or longitudinal).

5a Describe the questionnaire (e.g., number of sections, number of *6*

questions, number and names of instruments used).

Describe all questionnaire instruments that were used in the survey to *5*

Data collection methods

5b measure particular concepts. Report target population, reported validity and reliability information, scoring/classification procedure, and

reference links (if any).

Provide information on pretesting of the questionnaire, if performed (in the article or in an online supplement). Report the method of pretesting,

5c number of times questionnaire was pre-tested, number and demographics of participants used for pretesting, and the level of similarity of demographics between pre-testing participants and sample population.

*N.A.*

Sample characteristics

5d Questionnaire if possible, should be fully provided (in the article, or as appendices or as an online supplement).

6a Describe the study population (i.e., background, locations, eligibility criteria for participant inclusion in survey, exclusion criteria).

*46-54*

*5*

Describe the sampling techniques used (e.g., single stage or multistage *5*

6b sampling, simple random sampling, stratified sampling, cluster sampling, convenience sampling). Specify the locations of sample participants

whenever clustered sampling was applied.

6c Provide information on sample size, along with details of sample size calculation.

6d Describe how representative the sample is of the study population (or target population if possible), particularly for population-based surveys.

Provide information on modes of questionnaire administration, including

*5-6*

*5*

*5-6*

7a the type and number of contacts, the location where the survey was conducted (e.g., outpatient room or by use of online tools, such as

SurveyMonkey).

Survey administration

7b Provide information of survey’s time frame, such as periods of recruitment, exposure, and follow-up days.

Provide information on the entry process:

–>For non-web-based surveys, provide approaches to minimize human

*5-6*

7c error in data entry.

*6*

–>For web-based surveys, provide approaches to prevent “multiple participation” of participants.

Study preparation 8 Describe any preparation process before conducting the survey (e.g.,

interviewers’ training process, advertising the survey).

Provide information on ethical approval for the survey if obtained,

*N.A.*

*5*

Ethical considerations

including informed consent, institutional review board [IRB] approval, 9a Helsinki declaration, and good clinical practice [GCP] declaration (as

appropriate).

9b Provide information about survey anonymity and confidentiality and describe what mechanisms were used to protect unauthorized access.

10a Describe statistical methods and analytical approach. Report the statistical software that was used for data analysis.

10b Report any modification of variables used in the analysis, along with reference (if available).

Report details about how missing data was handled. Include rate of missing items, missing data mechanism (i.e., missing completely at

*5*

*6-7*

*N.A. 7*

Statistical analysis

10c

random [MCAR], missing at random [MAR] or missing not at random [MNAR]) and methods used to deal with missing data (e.g., multiple imputation).

10d State how non-response error was addressed. *N.A.*

10e For longitudinal surveys, state how loss to follow-up was addressed. *N.A.*

10f Indicate whether any methods such as weighting of items or propensity scores have been used to adjust for non-representativeness of the sample.

*N.A.*

10g Describe any sensitivity analysis conducted. *N.A.*

**Results**

11a Report numbers of individuals at each stage of the study. Consider using a flow diagram, if possible.

*N.A.*

Respondent

11b Provide reasons for non-participation at each stage, if possible. *N.A.*

characteristics

11c Report response rate, present the definition of response rate or the formula used to calculate response rate.

Provide information to define how unique visitors are determined. Report

*8*

*N.A.*

Descriptive

11d

number of unique visitors along with relevant proportions (e.g., view proportion, participation proportion, completion proportion).

Provide characteristics of study participants, as well as information on

*8-9*

12

results

potential confounders and assessed outcomes.

13a Give unadjusted estimates and, if applicable, confounder-adjusted estimates along with 95% confidence intervals and p-values.

For multivariable analysis, provide information on the model building

*10-11, 15*

*N.A.*

Main findings

**Discussion**

13b

13c

process, model fit statistics, and model assumptions (as appropriate).

Provide details about any sensitivity analysis performed. If there are considerable amount of missing data, report sensitivity analyses comparing the results of complete cases with that of the imputed dataset (if possible).

Discuss the limitations of the study, considering sources of potential

*N.A.*

*22-23*

Limitations 14

biases and imprecisions, such as non-representativeness of sample, study design, important uncontrolled confounders.

Interpretations 15 Give a cautious overall interpretation of results, based on potential biases and imprecisions and suggest areas for future research.

*24-25*

Generalizability 16 Discuss the external validity of the results. *22-23*

**Other sections**

Role of funding source

17 State whether any funding organization has had any roles in the survey’s design, implementation, and analysis.

*N.A.*

Conflict of interest 18 Declare any potential conflict of interest. *N.A.*

Acknowledgements 19 Provide names of organizations/persons that are acknowledged along *25*

with their contribution to the research.

Supplementary Table 22: Full questionnaire (German version)

# Zusätzliche Angaben zu Ihrer ärztlichen Tätigkeit

1. Bitte tragen Sie hier Ihre Sentinella-Nummer ein *****
2. Dispensieren Sie selbst Medikamente? *****
   1. **Ja**
   2. **Nein**
3. Nehmen Sie an Balint-Gruppen teil? *****
   1. **Ja**
   2. **Nein**
4. Haben Sie als Assistenzärzt*in eine psychiatrische Weiterbildung absolviert? *****
   1. **Ja (Assistenzstelle, psychiatrische Klinik/Poliklinik)**
   2. **Nein**
5. Interdisziplinärer Schwerpunkt Psychosomatische und Psychosoziale Medizin SAPPM: Haben Sie diesen Fähigkeitsausweis erlangt? *****
   1. **Ja**
   2. **Nein, aber ich befinde mich auf dem Weg dazu**
   3. **Nein**
6. Benutzen Sie Guidelines zur Abklärung und/oder für das Management von

depressiven Patienten und/oder Suizidalität? *****

- 1. **Nein**
  2. **Ja, nämlich…**

# Herangehensweise

1. Führen Sie in Ihrer Praxis ein systematisches (bei *jedem* Patienten/bei *jeder* Patientin), standardisiertes Depressionsscreening durch? *****
   1. **Ja**
   2. **Nein**
2. Wie diagnostizieren Sie eine Depression? (eine oder mehrere mögliche Antworten) *****
   1. **Diagnosemanual (ICD-10, ICD-11, DSM-5)**
   2. **PHQ-2**
   3. **PHQ-9**
   4. **Hamilton-Skala**
   5. **Beck (Beck-Depressions-Inventar)**
   6. **Andere…**
   7. **Ohne besondere diagnostische Mittel**
   8. **Bei Verdacht überweise ich diese Patienten**
3. Bei depressiven Patient*innen: Wie oft holen Sie Informationen von Familienangehörigen ein? *****
   1. **Nie (0-20%)**
   2. **Selten (21-40%)**
   3. **Manchmal (41-60%)**
   4. **Oft (61-80%)**
   5. **Immer (81-100%)**
4. Wie oft fragen Sie bei depressiven Patient*innen nach Angstsymptomen? *****
   1. **Nie (0-20%)**
   2. **Selten (21-40%)**
   3. **Manchmal (41-60%)**
   4. **Oft (61-80%)**
   5. **Immer (81-100%)**
5. Wie oft fragen Sie bei depressiven Patient*innen nach Schlafstörungen? *****
   1. **Nie (0-20%)**
   2. **Selten (21-40%)**
   3. **Manchmal (41-60%)**
   4. **Oft (61-80%)**
   5. **Immer (81-100%)**
6. Wie wichtig ist Ihnen die Abgrenzung einer Depression von bipolaren Störungen? *****
   1. **Völlig unwichtig**
   2. **Eher unwichtig**
   3. **Neutral**
   4. **Eher wichtig**
   5. **Sehr wichtig**
7. Wie oft fragen Sie bei depressiven Patient*innen nach Alkohol- und/oder Drogenkonsum? *****
   1. **Nie (0-20%)**
   2. **Selten (21-40%)**
   3. **Manchmal (41-60%)**
   4. **Oft (61-80%)**
   5. **Immer (81-100%)**

# Management

1. Ich fühle mich in der hausärztlichen Gesprächstherapie

(00.0520 Psychotherapeutische/psychosoziale Beratung durch den Facharzt/ die Fachärztin für Grundversorgung, pro 5 Min.)… *****

- 1. **Sehr kompetent**
  2. **Eher kompetent**
  3. **Neutral**
  4. **Eher inkompetent**
  5. **Sehr inkompetent**

1. Ich fühle mich in der Verschreibung einer medikamentösen antidepressiven Therapie… *****
   1. **Sehr kompetent**
   2. **Eher kompetent**
   3. **Neutral**
   4. **Eher inkompetent**
   5. **Sehr inkompetent**
2. Aus welchem Grund überweisen Sie depressive Patient*innen zur Psychotherapie? (eine oder mehrere mögliche Antworten) *****
   1. **Schweregrad der Depression**
   2. **Patientenwunsch**
   3. **Zeitmangel**
   4. **Zur eigenen emotionalen Entlastung**
   5. **Mangelndes Interesse**
   6. **Andere Gründe**
3. Aus welchem Grund verschreiben Sie depressiven Patient*innen Psychopharmaka? (eine oder mehrere mögliche Antworten) *****
   1. **Schweregrad der Depression**
   2. **Patientenwunsch**
   3. **Zeitmangel**
   4. **Begleitsymptome wie Angst oder Schlafstörungen**
   5. **Andere Gründe**
4. Fragen Sie nach Suizidalität bei depressiven Patient*innen? *****
   1. **Nie (0-20%)**
   2. **Selten (21-40%)**
   3. **Manchmal (41-60%)**
   4. **Oft (61-80%)**
   5. **Immer (81-100%)**
5. Welches ist der häufigste Grund/sind die häufigsten Gründe für eine dringliche psychiatrische Hospitalisierung? (eine oder mehrere mögliche Antworten) *****
   1. **Eigengefährdung/akute Suizidalität**
   2. **Psychiatrische Komorbidität, z.B. Suchtproblematik**
   3. **Patientenwunsch**
   4. **Angehörigenwunsch**
   5. **Schweregrad der Depression**
   6. **Fehlendes Ansprechen auf bisherige Behandlung**
   7. **Andere Gründe**
6. Wie oft geben Sie Ihren depressiven Patient*innen eine Notfalltelefonnummer mit (z.B. Triagenummer oder eigene Handynummer)? *****
   1. **Nie (0-20%)**
   2. **Selten (21-40%)**
   3. **Manchmal (41-60%)**
   4. **Oft (61-80%)**
   5. **Immer (81-100%)**

# Therapie

|  |  | **Nie (0-**  **20%)** | **Selten (21-**  **40%)** | **Manchmal**  **(41-60%)** | **Oft (61-**  **80%)** | **Immer (81-**  **100%)** |
| --- | --- | --- | --- | --- | --- | --- |
|  |  |  |  |  |  |  |
|  |  |  |  |  |  |  |
| 20) | Wie oft verschreiben Sie **Psychopharmaka** bei leichter Depression (ohne Angstsymptomatik/ohne Schlafstörungen)? ***** |  |  |  |  |  |
|  |  |  |  |  |  |  |
|  |  |  |  |  |  |  |
| 21) | Wie oft verschreiben Sie **Psychopharmaka** bei leichter Depression mit zusätzlicher Angstsymptomatik? ***** |  |  |  |  |  |
|  |  |  |  |  |  |  |
|  |  |  |  |  |  |  |
| 22) | Wie oft verschreiben Sie **Psychopharmaka** bei leichter Depression mit Schlafstörungen? ***** |  |  |  |  |  |
|  |  |  |  |  |  |  |
|  |  |  |  |  |  |  |
| 23) | Wie oft verschreiben Sie **andere Therapien (Bücher, Apps, "self-help", etc.)** bei leichter Depression (ohne Angstsymptomatik/ohne Schlafstörungen)? ***** |  |  |  |  |  |
|  |  |  |  |  |  |  |

| 24) | Wie oft verschreiben Sie **andere Therapien (Bücher, Apps, "self-help", etc.)** bei leichter  Depression mit zusätzlicher Angstsymptomatik? ***** |  |  |  |  |  |
| --- | --- | --- | --- | --- | --- | --- |
|  |  |  |  |  |  |  |
|  |  |  |  |  |  |  |
| 25) | Wie oft verschreiben Sie **andere Therapien (Bücher, Apps, "self-help", etc.)** bei leichter Depression mit Schlafstörungen? ***** |  |  |  |  |  |
|  |  |  |  |  |  |  |
|  |  |  |  |  |  |  |

1. Verfügbarkeit von Therapieplätzen bei **psychologischen Psychotherapeut*innen**: Wie einfach oder schwierig ist es einen Therapieplatz zu organisieren? *****
   1. **Sehr einfach**
   2. **Eher einfach, manchmal mit etwas Zeitaufwand verbunden**
   3. **Neutral**
   4. **Eher schwierig, grosser Zeitaufwand**
   5. **Sehr schwierig, sehr grosser Zeitaufwand**
2. Verfügbarkeit von Therapieplätzen bei **Psychiater*innen**: Wie einfach oder schwierig ist es einen Therapieplatz zu organisieren? *****
   1. **Sehr einfach**
   2. **Eher einfach, manchmal mit etwas Zeitaufwand verbunden**
   3. **Neutral**
   4. **Eher schwierig, grosser Zeitaufwand**
   5. **Sehr schwierig, sehr grosser Zeitaufwand**

# Interprofessionelle Zusammenarbeit/Kommunikation

1. Wie oft erhalten Sie von psychologischen Psychotherapeut*innen Rückmeldungen nach Überweisungen depressiver Patient*innen? *****
   1. **Nie (0-20%)**
   2. **Selten (21-40%)**
   3. **Manchmal (41-60%)**
   4. **Oft (61-80%)**
   5. **Immer (81-100%)**
2. Wie oft erhalten Sie von Psychiater*innen Rückmeldungen nach Überweisungen depressiver Patient*innen? *****
   1. **Nie (0-20%)**
   2. **Selten (21-40%)**
   3. **Manchmal (41-60%)**
   4. **Oft (61-80%)**
   5. **Immer (81-100%)**
3. In welcher Form erhalten Sie meist Rückmeldungen zur Diagnostik und/oder zum Therapieverlauf? *****
   1. **Brief per Post**
   2. **E-Mail**
   3. **Telefon**
   4. **Anderes…**
4. In welcher Form würden Sie gerne Rückmeldungen zur Diagnostik und/oder zum Therapieverlauf erhalten? *****
   1. **Brief per Post**
   2. **E-Mail**
   3. **Telefon**
   4. **Anderes…**

Supplementary Table 23: Full questionnaire (French version)

# Informations complémentaires sur votre activité médicale

1. Veuillez saisir votre numéro Sentinella ici *****
2. Dispensez-vous vous-même des médicaments? *****
   1. **Oui**
   2. **Non**
3. Participez-vous à des groupes Balint? *****
   1. **Oui**
   2. **Non**
4. En tant que médecin assistant avez-vous suivi une formation postgraduée en psychiatrie? *
   1. **Oui (poste d’assistant, clinique/policlinique psychiatrique)**
   2. **Non**
5. Formation approfondie interdisciplinaire Médecine Psychosomatique et Psychosociale ASMPP:

Avez-vous obtenu cette attestation? *****

- 1. **Oui**
  2. **Non, mais je suis sur le point de l’obtenir**
  3. **Non**

1. Utilisez-vous des lignes directrices pour l’examen et/ou pour la prise en charge des patients dépressifs et/ou suicidaires? *****
   1. **Non**
   2. **Oui, à savoir…**

# Approche

1. Effectuez-vous dans votre cabinet (pour *chaque* patient/patiente) un dépistage systématique et standardisé de la dépression? *****
   1. **Oui**
   2. **Non**
2. Comment diagnostiquez-vous une dépression ? (une ou plusieurs réponses possibles) *****
   1. **Manuel de diagnostic (ICD-10, ICD-11, DSM-5)**
   2. **PHQ-2**
   3. **PHQ-9**
   4. **Échelle de Hamilton**
   5. **Beck (Inventaire de dépression de Beck)**
   6. **Autres…**
   7. **Sans moyens diagnostiques particuliers**
   8. **En cas de suspicion, je transfère ces patients**
3. À quelle fréquence demandez-vous des informations aux membres de la famille des patients dépressifs? *
   1. **Jamais (0-20%)**
   2. **Rarement (21-40%)**
   3. **Parfois (41-60%)**
   4. **Souvent (61-80%)**
   5. **Toujours (81-100%)**
4. À quelle fréquence demandez-vous aux patients dépressifs s'ils présentent des symptômes d'anxiété? *
   1. **Jamais (0-20%)**
   2. **Rarement (21-40%)**
   3. **Parfois (41-60%)**
   4. **Souvent (61-80%)**
   5. **Toujours (81-100%)**
5. À quelle fréquence demandez-vous aux patients dépressifs s'ils ont des troubles du sommeil? *****
   1. **Jamais (0-20%)**
   2. **Rarement (21-40%)**
   3. **Parfois (41-60%)**
   4. **Souvent (61-80%)**
6. **Toujours (81-100%)**
7. Quelle importance accordez-vous à la distinction entre une dépression et un trouble bipolaire? *****
   1. **Totalement sans importance**
   2. **Plutôt sans importance**
   3. **Neutre**
   4. **Plutôt importante**
   5. **Très importante**
8. À quelle fréquence demandez-vous aux patients dépressifs s'ils consomment de l'alcool et/ou des drogues? *****
   1. **Jamais (0-20%)**
   2. **Rarement (21-40%)**
   3. **Parfois (41-60%)**
   4. **Souvent (61-80%)**
   5. **Toujours (81-100%)**

# Prise en charge

1. Dans la thérapie par le dialogue de médecin de famille

(00.0520 consultation psychothérapeutique ou psychosociale par le ou la médecin spécialiste de premier recours, par période de 5 min.) je me sens… *****

- 1. **très compétent/e**
  2. **plutôt compétent/e**
  3. **neutre**
  4. **plutôt incompétent/e**
  5. **très incompétent/e**

1. Dans la prescription d’un traitement médicamenteux antidépresseur, je me sens… *****
2. **très compétent/e**
3. **plutôt compétent/e**
4. **neutre**
5. **plutôt incompétent/e**
6. **très incompétent/e**
7. Pour quelle raison transférez-vous des patients dépressifs à une psychothérapie? (une ou plusieurs réponses possibles) *
   1. **Degré de gravité de la dépression**
   2. **Souhait du patient**
   3. **Manque de temps**
   4. **Pour se décharger émotionnellement**
   5. **Manque d’intérêt**
   6. **Autres raisons**
8. Pour quelle raison prescrivez-vous des psychotropes aux patients dépressifs? (une ou plusieurs réponses possibles) *****
   1. **Degré de gravité de la dépression**
   2. **Souhait du patient**
   3. **Manque de temps**
   4. **Symptômes associés tels que l’anxiété ou les troubles du sommeil**
   5. **Autres raisons**
9. Demandez-vous aux patients dépressifs s’ils ont des tendances suicidaires? *****
   1. **Jamais (0-20%)**
   2. **Rarement (21-40%)**
   3. **Parfois (41-60%)**
   4. **Souvent (61-80%)**
   5. **Toujours (81-100%)**
10. Quel est le motif le plus fréquent d'hospitalisation psychiatrique urgente ? (une ou plusieurs réponses possibles) *
    1. **Mise en danger de soi-même/tendances suicidaires aiguës**
    2. **Comorbidité psychiatrique, par ex. problème de dépendance**
    3. **Souhait du patient**
    4. **Souhait des proches**
    5. **Degré de gravité de la dépression**
    6. **Absence de réponse au traitement suivi**
    7. **Autres raisons**
11. À quelle fréquence donnez-vous un numéro de téléphone d'urgence à vos patients dépressifs? (par ex. numéro de tri ou numéro de portable personnel)? *
    1. **Jamais (0-20%)**
    2. **Rarement (21-40%)**
    3. **Parfois (41-60%)**
    4. **Souvent (61-80%)**
    5. **Toujours (81-100%)**

# Thérapie

|  |  |  | **Jamais (0-**  **20%)** | **Rarement**  **(21-**  **40%)** | **Parfois**  **(41-60%)** | **Souvent**  **(61-**  **80%)** | **Toujours**  **(81-**  **100%)** |
| --- | --- | --- | --- | --- | --- | --- | --- |
|  | | |  |  |  |  |  |

| 20) | À quelle fréquence prescrivez-vous des **psychotropes** en cas de dépression légère (sans symptôme d'anxiété/sans troubles du sommeil)? * |  |  |  |  |  |
| --- | --- | --- | --- | --- | --- | --- |
|  |  |  |  |  |  |  |
|  |  |  |  |  |  |  |
| 21) | À quelle fréquence prescrivez-vous des **psychotropes** en cas de dépression légère avec symptômes d'anxiété supplémentaires? * |  |  |  |  |  |
|  |  |  |  |  |  |  |
|  |  |  |  |  |  |  |
| 22) | À quelle fréquence prescrivez-vous des **psychotropes** en cas de dépression légère avec troubles du sommeil? * |  |  |  |  |  |
|  |  |  |  |  |  |  |
|  |  |  |  |  |  |  |
| 23) | À quelle fréquence prescrivez-vous d’**autres**  **thérapies (livres, apps, "self-help", etc.)** en cas de dépression légère (sans symptômes d’anxiété/sans troubles du sommeil)? ***** |  |  |  |  |  |
|  |  |  |  |  |  |  |
| 24) | À quelle fréquence prescrivez-vous d’**autres**  **thérapies (livres, apps, "self-help", etc.)** en cas de dépression légère avec symptômes d’anxiété supplémentaires? ***** |  |  |  |  |  |
|  |  |  |  |  |  |  |
| 25) | À quelle fréquence prescrivez-vous d’**autres**  **thérapies (livres, apps, "self-help", etc.)** en cas de dépression légère avec troubles du sommeil? ***** |  |  |  |  |  |
|  |  |  |  |  |  |  |
|  |  |  |  |  |  |  |

1. Disponibilité de places de thérapie chez les **psychothérapeutes psychologues**: est-il facile ou difficile d'organiser une place en thérapie? *
   1. **Très facile**
   2. **Plutôt facile, parfois lié à un certain investissement en temps**
   3. **Neutre**
   4. **Plutôt difficile, grand investissement de temps**
   5. **Très difficile, très grand investissement de temps**
2. Disponibilité de places de thérapie chez les **psychiatres**: est-il facile ou difficile d'organiser une place en thérapie? *
   1. **Très facile**
   2. **Plutôt facile, parfois lié à un certain investissement de temps**
   3. **Neutre**
   4. **Plutôt difficile, grand investissement de temps**
   5. **Très difficile, très grand investissement de temps**

# Collaboration interprofessionnelle/communication

1. À quelle fréquence recevez-vous des retours d’information de la part des psychothérapeutes psychologues après avoir transféré des patients dépressifs? *
   1. **Jamais (0-20%)**
   2. **Rarement (21-40%)**
   3. **Parfois (41-60%)**
   4. **Souvent (61-80%)**
   5. **Toujours (81-100%)**
2. À quelle fréquence recevez-vous des retours d’information de la part des psychiatres après avoir transféré des patients dépressifs? *
   1. **Jamais (0-20%)**
   2. **Rarement (21-40%)**
   3. **Parfois (41-60%)**
   4. **Souvent (61-80%)**
   5. **Toujours (81-100%)**
3. Sous quelle forme recevez-vous le plus souvent des retours d’information sur le diagnostic et/ou le déroulement de la thérapie? *
   1. **Lettre par courrier**
   2. **E-mail**
   3. **Téléphone**
   4. **Autres…**
4. Sous quelle forme aimeriez-vous recevoir des retours d’information sur le diagnostic et/ou le déroulement de la thérapie? *
   1. **Lettre par courrier**
   2. **E-mail**
   3. **Téléphone**
   4. **Autres…**

Supplementary Table 24: Full questionnaire (English version)

# Additional Information About Your Medical Practice

0) Please enter your Sentinella number *****

1. Do you dispense medications yourself? *****
   1. **Yes**
   2. **No**
2. Do you participate in Balint groups? *****
   1. **Yes**
   2. **No**
3. During your time as a resident doctor, did you complete psychiatric training? *****
   1. **Yes (residency in a psychiatric clinic/polyclinic)**
   2. **No**
4. Interdisciplinary Focus on Psychosomatic and Psychosocial Medicine (SAPPM): Have you obtained this qualification? *****
   1. **Yes**
   2. **No, but I am in the process of obtaining it**
   3. **No**
5. Do you use guidelines for the assessment and/or management of patients with depression and/or suicidality? *****
   1. **No**
   2. **Yes, specifically…**

# Approach

1. Do you conduct a systematic, standardized depression screening for each patient in your practice?

*****

- 1. **Yes**
  2. **No**

1. How do you diagnose depression? (select one or more responses) *****
   1. **Diagnostic manual (ICD-10, ICD-11, DSM-5)**
   2. **PHQ-2**
   3. **PHQ-9**
   4. **Hamilton Scale**
   5. **Beck Depression Inventory**
   6. **Other…**
   7. **Without specific diagnostic tools**
   8. **I refer these patients upon suspicion**
2. For patients with depression: How often do you gather information from family members? *****
   1. **Never (0-20%)**
   2. **Rarely (21-40%)**
   3. **Sometimes (41-60%)**
   4. **Often (61-80%)**
   5. **Always (81-100%)**
3. How often do you ask patients with depression about anxiety symptoms? *****
   1. **Never (0-20%)**
   2. **Rarely (21-40%)**
   3. **Sometimes (41-60%)**
   4. **Often (61-80%)**
   5. **Always (81-100%)**
4. How often do you ask patients with depression about sleep disturbances? *****
   1. **Never (0-20%)**
   2. **Rarely (21-40%)**
   3. **Sometimes (41-60%)**
   4. **Often (61-80%)**
   5. **Always (81-100%)**
5. How important is it to you to differentiate between depression and bipolar disorders? *****
   1. **Completely unimportant**
   2. **Rather unimportant**
   3. **Neutral**
   4. **Rather important**
   5. **Very important**
6. How often do you ask patients with depression about alcohol and/or drug use? *****
   1. **Never (0-20%)**
   2. **Rarely (21-40%)**
   3. **Sometimes (41-60%)**
   4. **Often (61-80%)**
   5. **Always (81-100%)**

# Management

1. In general practice talk therapy (00.0520 Psychotherapeutic/psychosocial counseling by a general practitioner, per 5 minutes), I feel…*****
   1. **Very competent**
   2. **Rather competent**
   3. **Neutral**
   4. **Rather incompetent**
   5. **Very incompetent**
2. In prescribing medication-based antidepressant therapy, I feel…*
   1. **Very competent**
   2. **Rather competent**
   3. **Neutral**
   4. **Rather incompetent**
   5. **Very incompetent**
3. For what reasons do you refer patients with depression to psychotherapy? (select one or more responses) *****
   1. **Severity of depression**
   2. **Patient request**
   3. **Lack of time**
   4. **Own emotional relief**
   5. **Lack of interest**
   6. **Other reasons**
4. For what reasons do you prescribe psychotropic drugs to patients with depression? (select one or more responses) *****
   1. **Severity of depression**
   2. **Patient request**
   3. **Lack of time**
   4. **Additional symptoms such as anxiety or sleep disturbances**
   5. **Other reasons**
5. Do you ask patients with depression about suicidality? *****
   1. **Never (0-20%)**
   2. **Rarely (21-40%)**
   3. **Sometimes (41-60%)**
   4. **Often (61-80%)**
   5. **Always (81-100%)**
6. What is the main reason/ are the main reasons for an urgent psychiatric hospitalization? (select one or more responses) *****
   1. **Risk to self/acute suicidality**
   2. **Psychiatric comorbidity, e.g., substance use issues**
   3. **Patient request**
   4. **Family request**
   5. **Severity of depression**
   6. **Lack of response to prior treatment**
   7. **Other reasons**
7. How often do you provide patients with depression an emergency phone number (e.g., triage number or personal mobile number)? *****
   1. **Never (0-20%)**
   2. **Rarely (21-40%)**
   3. **Sometimes (41-60%)**
   4. **Often (61-80%)**
   5. **Always (81-100%)**

# Therapy

|  |  | **Never (0-**  **20%)** | **Rarely (21-**  **40%)** | **Sometimes (41-60%)** | **Often (61-**  **80%)** | **Always (81-**  **100%)** |
| --- | --- | --- | --- | --- | --- | --- |
|  |  |  |  |  |  |  |
|  |  |  |  |  |  |  |
|  |  |  |  |  |  |  |
| 20) | How often do you prescribe **psychotropic drugs** for mild depression (without anxiety symptoms/without sleep disturbances)? ***** |  |  |  |  |  |
|  |  |  |  |  |  |  |
|  |  |  |  |  |  |  |
| 21) | How often do you prescribe **psychotropic drugs** for mild depression with additional anxiety symptoms?  ***** |  |  |  |  |  |
|  |  |  |  |  |  |  |
|  |  |  |  |  |  |  |
| 22) | How often do you prescribe **psychotropic drugs** for mild depression with sleep disturbances? ***** |  |  |  |  |  |
|  |  |  |  |  |  |  |
|  |  |  |  |  |  |  |
| 23) | How often do you prescribe **other therapies (books, apps, "self-help", etc.)** for mild depression (without anxiety symptoms/without sleep disturbances)? ***** |  |  |  |  |  |
|  |  |  |  |  |  |  |
| 24) | How often do you prescribe **other therapies (books, apps, "self-help", etc.)** for mild depression with additional anxiety symptoms? * |  |  |  |  |  |
|  |  |  |  |  |  |  |
|  |  |  |  |  |  |  |
| 25) | How often do you **other therapies (books, apps, "self-help", etc.)** for mild depression with sleep disturbances? * |  |  |  |  |  |
|  |  |  |  |  |  |  |
|  |  |  |  |  |  |  |

1. Availability of therapy places with **psychological psychotherapists**: How easy or difficult is it to arrange a therapy slot? *****
   1. **Very easy**
   2. **Rather easy, sometimes requiring some time investment**
   3. **Neutral**
   4. **Rather difficult, requiring a large time investment**
   5. **Very difficult, requiring a very large time investment**
2. Availability of therapy places with **psychiatrists**: How easy or difficult is it to arrange a therapy slot? *****
   1. **Very easy**
   2. **Rather easy, sometimes requiring some time investment**
   3. **Neutral**
   4. **Rather difficult, requiring a large time investment**
   5. **Very difficult, requiring a very large time investment**

# Interprofessional Collaboration/Communication

1. How often do you receive feedback from **psychological psychotherapists** after referring patients with depression? *****
   1. **Never (0-20%)**
   2. **Rarely (21-40%)**
   3. **Sometimes (41-60%)**
   4. **Often (61-80%)**
   5. **Always (81-100%)**
2. How often do you receive feedback from **psychiatrists** after referring patients with depression? *****
   1. **Never (0-20%)**
   2. **Rarely (21-40%)**
   3. **Sometimes (41-60%)**
   4. **Often (61-80%)**
   5. **Always (81-100%)**
3. In what form do you usually receive feedback on diagnostic and/or therapy progress? *****
   1. **Letter by mail**
   2. **E-Mail**
   3. **Phone**
   4. **Other…**
4. In what form would you prefer to receive feedback on diagnostic and/or therapy progress? *****
   1. **Letter by mail**
   2. **E-Mail**
   3. **Phone**
   4. **Other…**
